# Whole-genome sequence-based analysis of thyroid function

**DOI:** 10.1038/ncomms6681

**Published:** 2015-03-06

**Authors:** Peter N. Taylor, Eleonora Porcu, Shelby Chew, Purdey J. Campbell, Michela Traglia, Suzanne J. Brown, Benjamin H. Mullin, Hashem A. Shihab, Josine Min, Klaudia Walter, Yasin Memari, Jie Huang, Michael R. Barnes, John P. Beilby, Pimphen Charoen, Petr Danecek, Frank Dudbridge, Vincenzo Forgetta, Celia Greenwood, Elin Grundberg, Andrew D. Johnson, Jennie Hui, Ee M. Lim, Shane McCarthy, Dawn Muddyman, Vijay Panicker, John R.B. Perry, Jordana T. Bell, Wei Yuan, Caroline Relton, Tom Gaunt, David Schlessinger, Goncalo Abecasis, Francesco Cucca, Gabriela L. Surdulescu, Wolfram Woltersdorf, Eleftheria Zeggini, Hou-Feng Zheng, Daniela Toniolo, Colin M. Dayan, Silvia Naitza, John P. Walsh, Tim Spector, George Davey Smith, Richard Durbin, J. Brent Richards, Serena Sanna, Nicole Soranzo, Nicholas J. Timpson, Scott G. Wilson, The UK0K Consortium, Saeed Al Turki, Carl Anderson, Richard Anney, Dinu Antony, Maria Soler Artigas, Muhammad Ayub, Senduran Balasubramaniam, Jeffrey C. Barrett, Inês Barroso, Phil Beales, Jamie Bentham, Shoumo Bhattacharya, Ewan Birney, Douglas Blackwood, Martin Bobrow, Elena Bochukova, Patrick Bolton, Rebecca Bounds, Chris Boustred, Gerome Breen, Mattia Calissano, Keren Carss, Krishna Chatterjee, Lu Chen, Antonio Ciampi, Sebhattin Cirak, Peter Clapham, Gail Clement, Guy Coates, David Collier, Catherine Cosgrove, Tony Cox, Nick Craddock, Lucy Crooks, Sarah Curran, David Curtis, Allan Daly, Aaron Day-Williams, Ian N.M. Day, Thomas Down, Yuanping Du, Ian Dunham, Sarah Edkins, Peter Ellis, David Evans, Sadaf Faroogi, Ghazaleh Fatemifar, David R. Fitzpatrick, Paul Flicek, James Flyod, A. Reghan Foley, Christopher S. Franklin, Marta Futema, Louise Gallagher, Matthias Geihs, Daniel Geschwind, Heather Griffin, Detelina Grozeva, Xueqin Guo, Xiaosen Guo, Hugh Gurling, Deborah Hart, Audrey Hendricks, Peter Holmans, Bryan Howie, Liren Huang, Tim Hubbard, Steve E. Humphries, Matthew E. Hurles, Pirro Hysi, David K. Jackson, Yalda Jamshidi, Tian Jing, Chris Joyce, Jane Kaye, Thomas Keane, Julia Keogh, John Kemp, Karen Kennedy, Anja Kolb-Kokocinski, Genevieve Lachance, Cordelia Langford, Daniel Lawson, Irene Lee, Monkol Lek, Jieqin Liang, Hong Lin, Rui Li, Yingrui Li, Ryan Liu, Jouko Lönnqvist, Margarida Lopes, Valentina Lotchkova, Daniel MacArthur, Jonathan Marchini, John Maslen, Mangino Massimo, Iain Mathieson, Gaëlle Marenne, Peter McGuffin, Andrew McIntosh, Andrew G. McKechanie, Andrew McQuillin, Sarah Metrustry, Hannah Mitchison, Alireza Moayyeri, James Morris, Francesco Muntoni, Kate Northstone, Michael O'Donnovan, Alexandros Onoufriadis, Stephen O'Rahilly, Karim Oualkacha, Michael J. Owen, Aarno Palotie, Kalliope Panoutsopoulou, Victoria Parker, Jeremy R. Parr, Lavinia Paternoster, Tiina Paunio, Felicity Payne, Olli Pietilainen, Vincent Plagnol, Lydia Quaye, Michael A. Quai, Lucy Raymond, Karola Rehnström, Brent Richards, Susan Ring, Graham R.S. Ritchie, Nicola Roberts, David B. Savage, Peter Scambler, Stephen Schiffels, Miriam Schmidts, Nadia Schoenmakers, Robert K. Semple, Eva Serra, Sally I. Sharp, So-Youn Shin, David Skuse, Kerrin Small, Lorraine Southam, Olivera Spasic-Boskovic, David St Clair, Jim Stalker, Elizabeth Stevens, Beate St Pourcian, Jianping Sun, Jaana Suvisaari, Ionna Tachmazidou, Martin D. Tobin, Ana Valdes, Margriet Van Kogelenberg, Parthiban Vijayarangakannan, Peter M. Visscher, Louise V. Wain, James T.R. Walters, Guangbiao Wang, Jun Wang, Yu Wang, Kirsten Ward, Elanor Wheeler, Tamieka Whyte, Hywel Williams, Kathleen A. Williamson, Crispian Wilson, Kim Wong, ChangJiang Xu, Jian Yang, Fend Zhang, Pingbo Zhang

**Affiliations:** 1 Thyroid Research Group, Institute of Molecular & Experimental Medicine, Cardiff University School of Medicine, Cardiff University, Cardiff, UK; 2 Istituto di Ricerca Genetica e Biomedica (IRGB), Consiglio Nazionale delle Ricerche, c/o Cittadella Universitaria di Monserrato, Monserrato, Cagliari, Italy; 3 Dipartimento di Scienze Biomediche, Università di Sassari, Sassari, Italy; 4 Center for Statistical Genetics, Biostatistics Department, University of Michigan, Ann Arbor, Michigan, USA; 5 Department of Endocrinology and Diabetes, Sir Charles Gairdner Hospital, Nedlands, Western Australia, Australia; 6 Division of Genetics and Cell Biology, San Raffaele Research Institute, Milano, Italy; 7 School of Medicine and Pharmacology, University of Western Australia, Crawley, Western Australia, Australia; 8 MRC Integrative Epidemiology Unit at the University of Bristol, University of Bristol, Bristol, UK; 9 Wellcome Trust Sanger Institute, Wellcome Trust Genome Campus, Cambridge, UK; 10 William Harvey Research Institute, Barts and The London School of Medicine and Dentistry, Queen Mary University of London, London, UK; 11 Pathwest Laboratory Medicine WA, Nedlands, Western Australia, Australia; 12 School of Pathology and Laboratory Medicine, University of Western Australia, Crawley, Western Australia, Australia; 13 Faculty of Epidemiology and Population Health, London School of Hygiene and Tropical Medicine, London, UK; 14 Department of Tropical Hygiene, Faculty of Tropical Medicine, Mahidol University, Bangkok, Thailand; 15 Lady Davis Institute, Jewish General Hospital, McGill University, Montréal, Québec, Canada; 16 Department of Oncology, Department of Epidemiology, Biostatistics and Occupational Health, Jewish General Hospital, Lady Davis Institute, McGill University, Montréal, Québec, Canada; 17 Department of Human Genetics, McGill University, Montreal, Québec, Canada H3A1A5; 18 McGill University and Genome Quebec Innovation Centre, Montreal, Québec, Canada H3A1A5; 19 Cardiovascular Epidemiology and Human Genomics Branch, National Heart, Lung and Blood Institute, Bethesda, Maryland, USA; 20 MRC Epidemiology Unit, University of Cambridge School of Clinical Medicine, Institute of Metabolic Science, Cambridge Biomedical Campus, Box 285, Cambridge, UK; 21 Department of Twin Research and Genetic Epidemiology, King's College London, London, UK; 22 Laboratory of Genetics, NIA, Baltimore, Maryland 21224, USA; 23 Facharzt für Laboratoriumsmedizin, Geschäftsführer amedes Ost, Halle/Leipzig GmbH, Leipziger Chaussee 191f, 06112 Halle (Saale), Germany; 24 Institute of Molecular Genetics–CNR, Pavia, Italy; 25 Department of Pathology, King Abdulaziz Medical City, Riyadh, Saudi Arabia; 26 Department of Psychiatry, Trinity Centre for Health Sciences, St James Hospital, James's Street, Dublin 8, Ireland; 27 Genetics and Genomic Medicine and Birth Defects Research Centre, UCL Institute of Child Health, London WC1N 1EH, UK; 28 Division of Developmental Disabilities, Department of Psychiatry, Queen's University, Kingston, Ontario, Canada; 29 University of Cambridge Metabolic Research Laboratories, NIHR Cambridge Biomedical Research Centre, Wellcome Trust-MRC Institute of Metabolic Science, Addenbrooke's Hospital, Cambridge CB2 0QQ, UK; 30 Department of Cardiovascular Medicine and Wellcome Trust Centre for Human Genetics, Roosevelt Drive, Oxford OX3 7BN, UK; 31 European Molecular Biology Laboratory, European Bioinformatics Institute, Wellcome Trust Genome Campus, Hinxton, Cambridge CB10 1SD, UK; 32 Division of Psychiatry, The University of Edinburgh, Royal Edinburgh Hospital, Edinburgh EH10 5HF, UK; 33 Department of Medical Genetics, Cambridge Institute for Medical Research, University of Cambridge, Cambridge CB2 0XY, UK; 34 Institute of Psychiatry, King's College London, 16 De Crespigny Park, London SE5 8AF, UK; 35 NIHR BRC for Mental Health, Institute of Psychiatry and SLaM NHS Trust, King's College London, 16 De Crespigny Park, London SE5 8AF, UK; 36 Dubowitz Neuromuscular Centre, UCL Institute of Child Health and Great Ormond Street Hospital, London WC1N 1EH, UK; 37 Department of Haematology, University of Cambridge, Long Road, Cambridge CB2 0PT, UK; 38 Department of Epidemiology, Biostatistics and Occupational Health, McGill University, Montreal, Quebec, Canada; 39 Institut für Humangenetik, Uniklinik Köln, Kerpener Strasse 34, 50931 Köln, Germany; 40 Social, Genetic and Developmental Psychiatry Centre, Institute of Psychiatry, King's College London, Denmark Hill, London SE5 8AF, UK; 41 Lilly Research Laboratories, Eli Lilly and Co. Ltd., Erl Wood Manor, Sunninghill Road, Windlesham, Surrey, UK; 42 MRC Centre for Neuropsychiatric Genetics and Genomics, Institute of Psychological Medicine and Clinical Neurosciences, School of Medicine, Cardiff University, Cardiff CF14 4XN, UK; 43 Sheffield Diagnostic Genetics Service, Sheffield Childrens' NHS Foundation Trust, Western Bank, Sheffield S10 2TH, UK; 44 University of Sussex, Brighton BN1 9RH, UK; 45 Sussex Partnership NHS Foundation Trust, Swandean, Arundel Road, Worthing, West Sussex BN13 3EP, UK; 46 University College London (UCL), Molecular Psychiatry Laboratory, Division of Psychiatry, Gower Street, London WC1E 6BT, UK; 47 Computational Biology and Genomics, Biogen Idec, 14 Cambridge Center, Cambridge, Massachusetts 02142, USA; 48 Division of Genetics and Molecular Medicine, Department of Medical and Molecular Genetics, King's College London School of Medicine, Guy's Hospital, London SE1 9RT, UK; 49 BGI-Shenzhen, Shenzhen 518083, China; 50 University of Queensland Diamantina Institute, Translational Research Institute, Brisbane, Queensland, Australia; 51 MRC Human Genetics Unit, MRC Institute of Genetics and Molecular Medicine, the University of Edinburgh, Western General Hospital, Edinburgh, EH4 2XU, UK; 52 The Genome Centre, John Vane Science Centre, Queen Mary, University of London, Charterhouse Square, London EC1M 6BQ, UK; 53 Cardiovascular Genetics, BHF Laboratories, Rayne Building, Institute Cardiovascular Sciences, University College London, London WC1E 6JJ, UK; 54 David Geffen School of Medicine, Los Angeles, California, USA; 55 Davis Institute, Jewish General Hospital, Montreal, Quebec, Canada; 56 Medicine and Human Genetics, McGill University, Montreal, Quebec, Canada; 57 Department of Oncology, McGill University, Montreal, Quebec, Canada; 58 HeLEX—Centre for Health, Law and Emerging Technologies, Department of Public Health, University of Oxford, Old Road Campus, Oxford OX3 7LF, UK; 59 Department of Mathematical and Statistical Sciences, University of Colorado, Denver, Colorado 80202, USA; 60 Adaptive Biotechnologies Corporation, Seattle, Washington, USA; 61 Human Genetics Research Centre, St George's University of London, UK; 62 Behavioural and Brain Sciences Unit, UCL Institute of Child Health, London WC1N 1EH, UK; 63 Analytic and Translational Genetics Unit, Massachusetts General Hospital, Boston, Massachusetts 02114, USA; 64 BGI-Europe, London, UK; 65 National Institute for Health and Welfare (THL), Helsinki, Finland; 66 Wellcome Trust Centre for Human Genetics, Roosevelt Drive, Oxford OX3 7BN, UK; 67 Program in Medical and Population Genetics and Genetic Analysis Platform, The Broad Institute of MIT and Harvard, Cambridge, Massachusetts 02132, USA; 68 Department of Statistics, University of Oxford, 1 South Parks Road, Oxford OX1 3TG, UK; 69 Department of Genetics, Harvard Medical School, Boston, Massachusetts 02115, USA; 70 The Patrick Wild Centre, The University of Edinburgh, Edinburgh EH10 5HF, UK; 71 The Department of Epidemiology and Biostatistics, Imperial College London, St.Mary's campus, Norfolk Place, Paddington, London W2 1PG, UK; 72 Department of Mathematics, Université de Québec À Montréal, Montréal, Québec, Canada; 73 Institute for Molecular Medicine Finland (FIMM), University of Helsinki, Helsinki, Finland; 74 Institute of Neuroscience, Henry Wellcome Building for Neuroecology, Newcastle University, Framlington Place, Newcastle upon Tyne NE2 4HH, UK; 75 Department of Psychiatry, University of Helsinki, Helsinki, Finland; 76 MRC Epidemiology Unit, Institute of Metabolic Science, Addenbrooke's Hospital, Box 285, Hills Road, Cambridge CB2 0QQ, UK; 77 University College London (UCL) Genetics Institute (UGI), Gower Street, London WC1E 6BT, UK; 78 ALSPAC School of Social and Community Medicine, University of Bristol, Oakfield House, Oakfield Grove, Clifton, Bristol BS8 2BN, UK; 79 Institute of Medical Sciences, University of Aberdeen, Aberdeen AB25 2ZD, UK; 80 School of Oral and Dental Sciences, University of Bristol, Lower Maudlin Street, Bristol BS1 2LY, UK; 81 School of Experimental Psychology, University of Bristol, 12a Priory Road, Bristol BS8 1TU, UK; 82 Queensland Brain Institute, University of Queensland, Brisbane, Queensland 4072, Australia; 83 Departments of Health Sciences and Genetics, University of Leicester, Leicester, UK; 84 Department of Medicine, State Key Laboratory of Pharmaceutical Biotechnology, University of Hong Kong, 21 Sassoon Road, Hong Kong, China; 85 Department of Biology, University of Copenhagen, Ole Maaløes Vej 5, 2200 Copenhagen, Denmark; 86 Princess Al Jawhara Albrahim Center of Excellence in the Research of Hereditary Disorders, King Abdulaziz University, Jeddah, Saudi Arabia; 87 Macau University of Science and Technology, Avenida Wai long, Taipa, Macau 999078, China; 88 Program in Medical and Population Genetics, Broad Institute of Harvard and MIT, Cambridge, Massachusetts 02132, USA

## Abstract

Normal thyroid function is essential for health, but its genetic architecture remains poorly understood. Here, for the heritable thyroid traits thyrotropin (TSH) and free thyroxine (FT4), we analyse whole-genome sequence data from the UK10K project (*N*=2,287). Using additional whole-genome sequence and deeply imputed data sets, we report meta-analysis results for common variants (MAF≥1%) associated with TSH and FT4 (*N*=16,335). For TSH, we identify a novel variant in *SYN2* (MAF=23.5%, *P*=6.15 × 10^−9^) and a new independent variant in *PDE8B* (MAF=10.4%, *P*=5.94 × 10^−14^). For FT4, we report a low-frequency variant near *B4GALT6/SLC25A52* (MAF=3.2%, *P*=1.27 × 10^−9^) tagging a rare *TTR* variant (MAF=0.4%, *P*=2.14 × 10^−11^). All common variants explain ≥20% of the variance in TSH and FT4. Analysis of rare variants (MAF<1%) using sequence kernel association testing reveals a novel association with FT4 in *NRG1.* Our results demonstrate that increased coverage in whole-genome sequence association studies identifies novel variants associated with thyroid function.

## Abstract (web summary)

Levels of circulating thyrotropin and free thyroxine reflect thyroid function, however, their genetic underpinnings remain poorly understood. Taylor *et al*. take advantage of whole-genome sequence data from cohorts within the UK10K project to identify novel variants associated with these traits.

## Graphical abstract

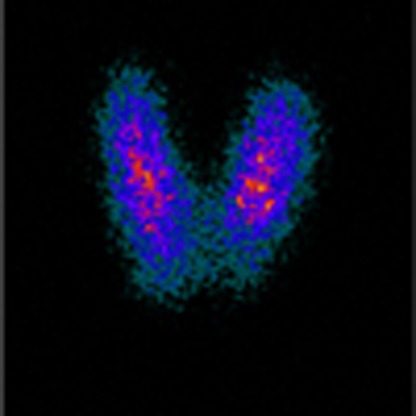

Thyroid hormones have fundamental but diverse physiological roles in vertebrate physiology, ranging from induction of metamorphosis in amphibians to photoperiodic regulation of seasonal breeding in birds^1^. In humans, they are essential for adult health and childhood development^2^,^3^ and levothyroxine is one of the commonest drugs prescribed worldwide. Clinically, thyroid function is assessed by measuring circulating concentrations of free thyroxine (FT4) and the pituitary hormone thyrotropin (TSH); the complex inverse relationship between them renders TSH the more sensitive marker of thyroid status^4^. Even small differences in TSH and FT4, within the normal population reference range, are associated with a wide range of clinical parameters, including blood pressure, lipids and cardiovascular mortality, as well as obesity, bone mineral density and lifetime cancer risk^5^.

Twin and family studies estimate the heritability of TSH and FT4 as up to 65%^6^. Genome-wide association studies (GWAS) identified common variants associated with TSH and FT4^7^,^8^,^9^; in a recent HapMap-based meta-analysis^10^, we identified 19 loci associated with TSH and 4 with FT4. However, these accounted for only 5.6% of the variance in TSH and 2.3% in FT4. Therefore, most of the heritability of these important traits remains unexplained.

The unidentified genetic component of variance might be explained by common variants poorly tagged by markers assessed in previous studies, or those with small effects. However, rarer variants within the minor allele frequency (MAF) spectrum might also account for a substantial proportion of the missing heritability as has been proposed for many polygenic traits^11^. These variants, although individually rare (MAF<1%), are collectively frequent, and while their effects may be insufficient to produce clear familial aggregation, effect sizes for individual variants are potentially much greater than those observed for common variants. In addition, a greater understanding of the relative proportion of thyroid function explained by common variants is now possible with the availability of whole-genome sequencing (WGS) and this is essential to refine future research and analysis strategies when appraising the genetic architecture of thyroid function.

In this study, the first to utilize WGS to examine the genetic architecture of TSH and FT4, we perform single-point association analysis in two discovery cohorts in the UK10K project with WGS data available and a meta-analysis using genome wide association data (GWAS) with deep imputation from five additional data sets. We report three new loci associated with thyroid function in healthy individuals, undertake quantitative trait loci and DNA methylation analyses to further study these relationships and undertake genome-wide complex trait analyses (GCTA)^12^ to assess the contributions of common variants (MAF≥1%) to variance in thyroid function. We also explore whether there is a shared polygenic basis between TSH and FT4. In individuals with WGS data, we perform sequence kernel-based association testing (SKAT) analysis to identify regions of the genome where rare variants have the strongest association with thyroid function and identify a novel locus associated with FT4. The results demonstrate that WGS-based analyses can identify rare functional variants and associations derived from rare aggregates. Larger meta-analyses of studies with WGS data are now required to identify additional common and rare variants, which may explain the missing heritability of thyroid function.

## Results

### Single-point association analysis

In the discovery study, using a meta-analysis of WGS data from the Avon Longitudinal Study of Parents and Children (ALSPAC) and TwinsUK cohorts (*N*=2,287) analysing up to 8,816,734 markers (Supplementary Tables 1 and 2; Supplementary Methods), we find associations at two previously described loci for TSH. These are *NR3C2* (rs11728154; MAF=21.0%, *B*=0.21, s.e.=0.037, *P*=8.21 × 10^−9^; *r*^2^=0.99 with the previously reported rs10028213) and *FOXE1* (rs1877431; MAF=39.5%, *B*=−0.19, s.e.=0.030, *P*=2.29 × 10^−10^; *r*^2^=0.99 with the previously reported rs965513). We find one borderline signal (between *P*=5.0 × 10^−08^ and *P*=1.17 × 10^−08^) at a novel locus *FAM222A* (rs11067829; MAF=18.3%, *B*=0.210, s.e.=0.038, *P*=3.73 × 10^−8^; Supplementary Figs 1a and 2; Supplementary Table 3). No variants show genome-wide significant association for FT4 (Supplementary Figs 1a and 3).

**Figure 1:**
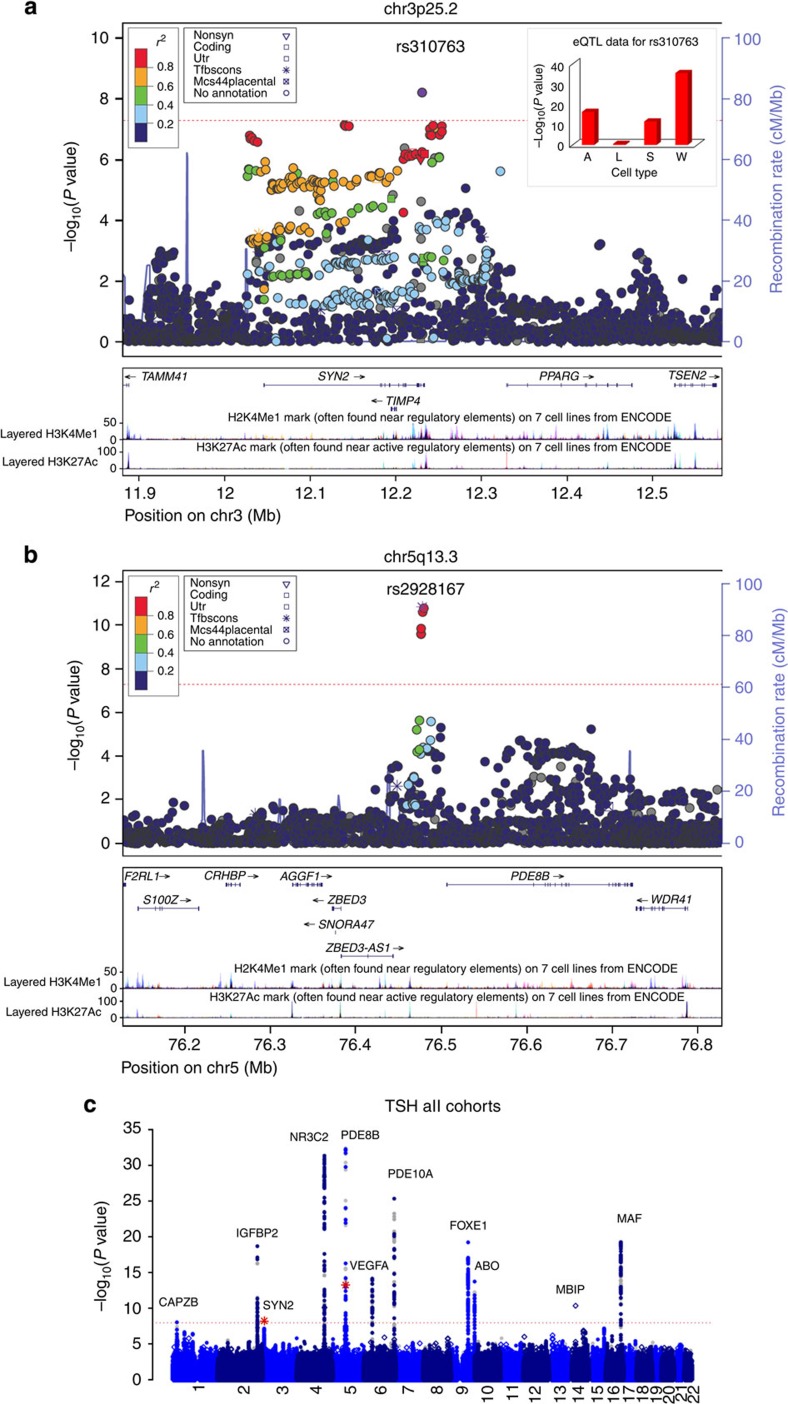
Regional and genome-wide association plots for TSH. (**a**) Regional association plot showing genome-wide significant locus for serum TSH at the *SYN2, TIMP4* gene region. Inset is *in vitro* expression QTL data for the lead SNP rs310763 in adipose cells (A), lymphoblastoid cell lines (L), skin cells (S) and whole blood (W). Dotted line denotes genome-wide significance threshold. (**b**) Regional association plot after conditional analysis on rs2046045 in *PDE8B* showing our novel association with TSH at rs2928167 in *PDE8B* remained genome-wide significant. (**c**) Annotated Manhattan plot from the overall analysis for serum TSH levels. SNPs (MAF>1%) are plotted on the *x* axis according to their position on each chromosome against association with TSH on the *y* axis (shown as −log_10_ (*P* value)). The loci are regarded as genome-wide significant at *P*<5 × 10^−8^. Variants with 1%<MAF<5% are shown as open diamond symbols. Common SNPs (MAF>5%) are shown as solid circles with those present in Hapmap II reference panels in grey and those derived from WGS or deeply imputed using WGS and 1000 genomes reference panels in blue. SNPs shown as a red asterisk represent novel genome-wide significant findings.

**Figure 2:**
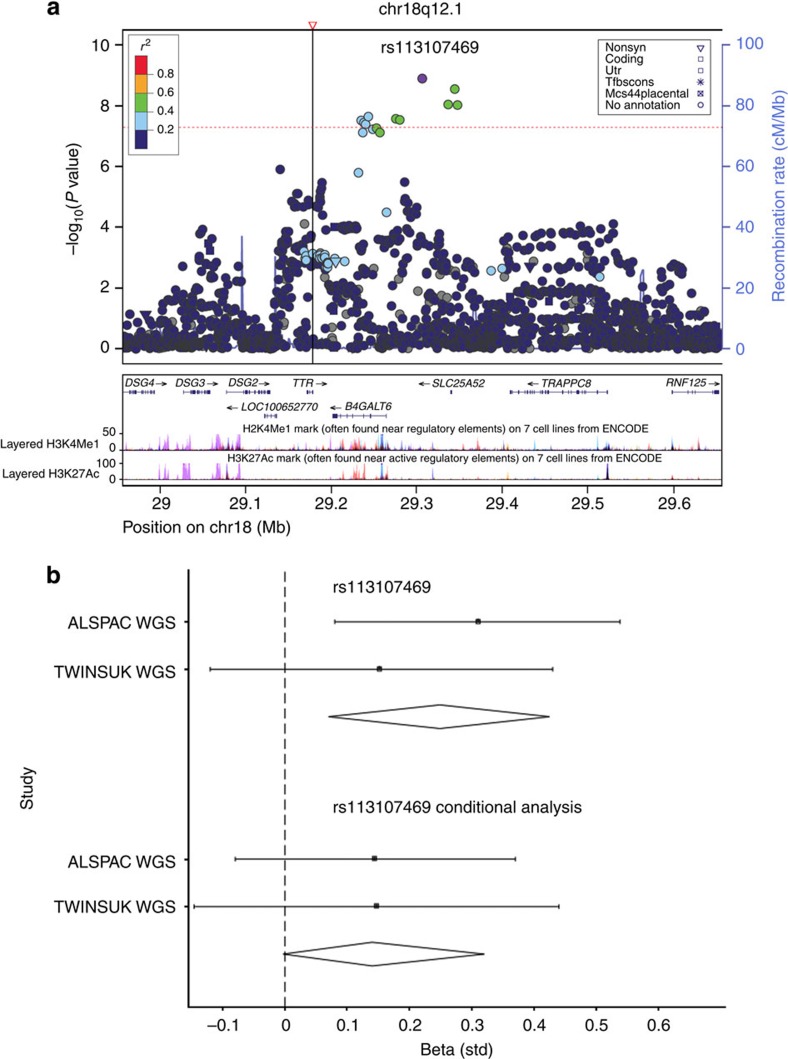
Regional and conditional plots for FT4. (**a**) Regional association plot showing genome-wide significant locus for serum FT4 at the *B4GALT6, SLC25A52* region (overall meta-analysis). ∇ shows the location of the Thr139Met substitution (rs28933981; MAF=0.4%) in *TTR*. Dotted red line denotes genome-wide significance threshold. (**b**) Forest plots of WGS association data for rs113107469 in the WGS discovery studies and meta-analysis, and below is the illustrating loss of signal on conditioning with rs28933981. Squares represent beta estimate and error bars represent 95% CI.

**Figure 3:**
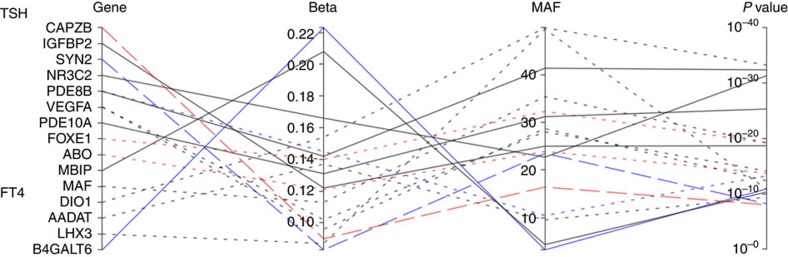
Overview of our findings of SNPs associated with TSH and FT4. Blue coloured lines represent a novel signal identified in this meta-analysis. Red lines represent heterogeneity observed between the different cohorts in the association between the variant and TSH. **- - -** Indicates responsiveness observed to levothyroxine. — — Indicates observed eQTL or meQTL associations.

In a meta-analysis of the discovery cohorts and five additional cohorts, we find associations for 13 SNPs at 11 loci for TSH (*N*=16,335) of which 11 loci have been identified previously and 4 SNPs at 4 loci for FT4 (*N*=13,651) of which 3 have been identified previously (Table 1; Figs 1a–c,2a,b and 3; Supplementary Figs 1b and 3–6).

To determine whether our identified associations at established loci represented previous association signals, we analysed the linkage disequilibrium (LD) between the strongest associated variants from this study and those from our previous study^10^ (Supplementary Table 4). The top variants from loci in both studies were in strong LD (*r*^2^>0.6), apart from *MBIP* and *FOXE1*, although these were in strong LD with variants previously associated with TSH by others^8^. Two SNPs associated with TSH in our study are novel, one at *SYN2* (rs310763; MAF=23.5%, *B*=0.082, s.e.=0.014, *P*=6.15 × 10^−9^; Fig. 1a–c). *SYN2* is a member of a family of neuron-specific phosphoproteins involved in the regulation of neurotransmitter release with expression in the pituitary and hypothalamus (http://biogps.org/#goto=genereport&id=6854). We also identify one novel variant at *PDE8B* (MAF=10.4%, *B*=−0.145, s.e.=0.019, *P*=5.94 × 10^−14^) in linkage equilibrium (*r*^2^=0.002, *D*′=0.17) with the previously described variant rs6885099 (ref. 10) and independent from our top SNP rs2046045 (*P*=1.93 × 10^−11^) after conditional analysis. In the overall meta-analysis, we are unable to replicate the association between *FAM222A* and TSH in the discovery analysis (*B*=0.014, s.e.=0.015, *P*=0.378); however, we observe evidence of heterogeneity between cohorts (test for heterogeneity *P*=4.70 × 10^−6^; Supplementary Table 5), so potentially this locus may find support in future WGS studies.

In our meta-analysis, we also identify four SNPs associated with FT4, three at previously established loci (*DIO1*, *LHX3* and *AADAT*; Table 1; Fig. 3; Supplementary Figs 1b, 4e and 6; Supplementary Table 4). We find a novel uncommon variant at *B4GALT6*/*SLC25A52* associated with FT4 (rs113107469; MAF=3.20%, *B*=0.225, s.e.=0.037, *P=*1.27 × 10^−9^; Fig. 2a). *B4GALT6* is in the ceramide metabolic pathway, which inhibits cyclic AMP production in TSH-stimulated cells. However, the *B4GALT6* signal (rs113107469) is in weak LD (*r*^2^<0.1, *D*′=0.66) with the Thr139Met substitution (rs28933981; MAF=0.4%) and it may therefore be a marker for this functional change in *TTR*. The Thr139Met substitution was associated with FT4 levels in our single-point meta-analysis (*P*=2.14 × 10^−11^), however, was not originally observed as the MAF was lower than our 1% threshold. Conditional analysis of the *TTR* region using rs28933981 as the conditioning marker in the ALSPAC WGS cohort reveals no evidence of association between rs113107469 in *B4GALT6* and FT4 (*P*=0.124; Fig. 2b). Analysis using direct genotyping in the ALSPAC WGS and replication cohorts confirms the effect of the Thr139Met substitution on FT4 levels. Here, 0.79% of children were heterozygous for the Thr139Met substitution, which is positively associated with FT4 (*B*=1.70, s.e.=0.17, 95% CI 1.37, 2.03, *P*=3.89 × 10^−24^). In the ALSPAC replication data set, rs113107469 in *B4GALT6* was also positively associated with FT4 (*P*=0.0002); however, when conditioned on the Thr139Met substitution there was no longer any evidence of association (*P*=0.20). The Thr139Met substitution also appears to be functional: this mutation has increased protein stability compared with wild-type transthyretin (TTR)^13^,^14^ and tighter binding of thyroxine^14^, resulting in a twofold increase in thyroxine-binding affinity^15^,^16^. Further details of the likely genes related to all our observed independent novel signals are shown in Supplementary Table 6.

### Expression quantitative trait locus analysis

Expression quantitative trait locus (eQTL) analysis^17^,^18^ reveals that our *SYN2* variant modulates *SYN2* transcription in adipose, skin and whole-blood cells, but not lymphoblastoid cell lines (Supplementary Table 7). Furthermore, bioinformatics analysis suggests that the C-allele at rs310763 attenuates an EGR1 regulatory motif^19^. *EGR1* is expressed in thyrocytes, regulates pituitary development^20^,^21^ and may influence thyroid status via *LHX3* promotor activity^20^. Several other variants in the *SYN2* gene region are in strong LD (*r*^2^>0.8) with rs310763, including the non-synonymous coding variant rs794999. Although predicted to be benign (PolyPhen-2 score=0.002 (ref. 22)), rs794999 is located in a DNase hypersensitivity cluster^23^, influences four predicted regulatory motifs^19^ and appears to be under evolutionary constraint^24^,^25^. SNPs identified in our study, or those in LD, also showed strong eQTL associations with *PDE8B* (*P*=8.69 × 10^−27^), *FOXE1* (*P=*9.10 × 10^−54^) and *AADAT* (*P*=7.86 × 10^−9^) gene expressions (Supplementary Table 7).

### DNA methylation analysis

To further explore *cis*-regulatory effects of variants identified in our study, we carried out analysis of DNA methylation profiles in whole-blood samples in 279 individuals from the TwinsUK cohort. We find evidence for a methylation quantitative trait locus (meQTL) at the novel TSH-associated variant rs2928167 in *PDE8B* (*P*=4.38 × 10^−7^; Supplementary Table 8), which are also eQTLs in multiple tissues (Supplementary Table 7). Recently, meQTL effects using the same probe (cg16418800) in adipose tissue also identified a peak signal at rs2359775 (*P*=6 × 10^−15^), which is in LD with rs2928167 (*r*^2^=0.5). We find that variants in *ABO* (*P*=2.02 × 10^−23^) and *AADAT* (*P*=1.80 × 10^−8^) also show strong evidence for *cis*-meQTL effects (Supplementary Table 8). In additional analyses in 745 ALSPAC children, we find strong meQTL associations for rs2359775 in *PDE8B* (*P*=3.03 × 10^−28^) and variants in *ABO* (*P*=1.01 × 10^−101^) and *AADAT* (*P*=4.18 × 10^−34^) (Supplementary Table 8).

### SKAT analysis

**Figure 4:**
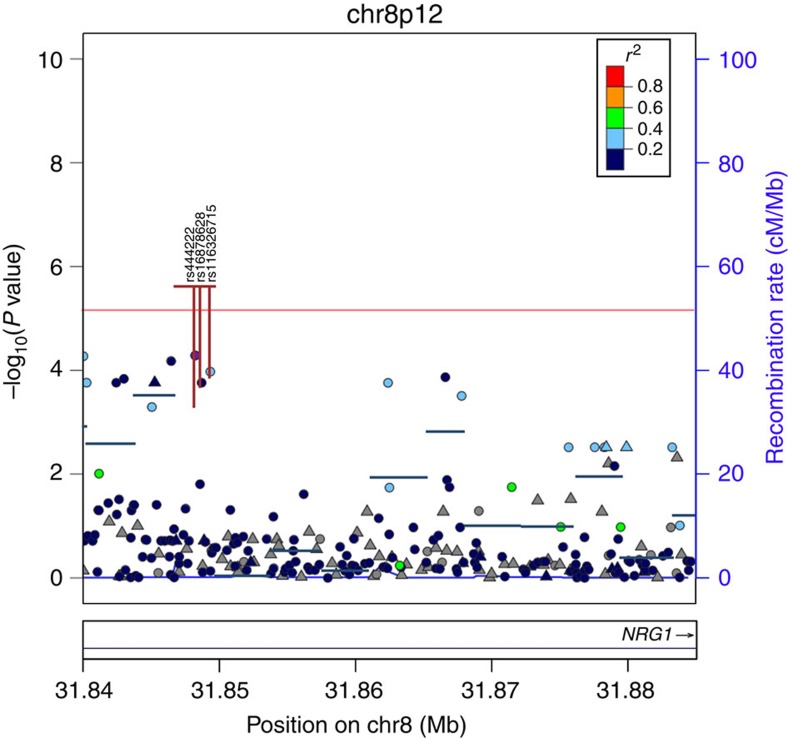
Plots showing *NRG1* region with significant associations with FT4 from SKAT analysis. Horizontal bar represents SKAT variant bins. (·)=single-point association data. Vertical lines in the bin (|) highlight rare variants that contribute to the association with a contribution proportional to the length of the line (that is, removal of the variant from the analysis causes the significance to fall to the level shown).

Tests of the association between aggregates of rare variants (MAF<1%) in the WGS cohorts were restricted to genes relevant to thyroid function. We find no evidence of association from SKAT analyses with TSH, however, for FT4 we identify one SKAT bin with multiple-testing-corrected evidence for association (*P*≤1.55 × 10^−5^) in *NRG1* (*P*=2.53 × 10^−6^; Fig. 4; Supplementary Table 9). NRG1 is a glycoprotein that interacts with the NEU/ERBB2 receptor tyrosine kinase, and is critical in organ development.

### GCTA and polygenic score analysis

SNPs were thinned to a set of 2,203,581 approximately independent SNPs with an LD threshold of *r*^2^>0.2, a window size of 5,000 SNPs and step of 1,000 SNPs. A genomic relationship matrix was then generated for unrelated individuals. We fitted linear mixed-effect models and estimate that all assessed common SNPs (MAF>1%) explain 24% (95% CI 19, 29) and 20% (95% CI 14, 26) of TSH and FT4 variance, respectively (*P*≤0.0001; Supplementary Table 10). Polygenic score analyses^21^ based on SNPs with *P* values under a fixed threshold do not detect evidence of a polygenic signal for TSH or FT4, nor of a shared polygenic basis between thyroid function and key metabolic outcomes. However, a genetic score based on 67 SNPs previously associated with thyroid function in GWAS^8^,^10^,^26^ shows strong evidence of association with TSH (*P*=7.9 × 10^−20^) and FT4 (*P*=2.7 × 10^−4^) and we observe evidence of shared genetic pathways with TSH associated with the FT4 gene score (*P*=7.0 × 10^−4^). These 67 SNPs explain 7.1% (95% CI 5.2, 9.0) of the variance in TSH and 1.9% (95% CI 1.1, 3.0) of the variance in FT4. Taken together, this suggests that many loci underlying thyroid function remain unknown.

### Chemogenomic analysis

We undertook a database analysis of differential gene expression in cultured cells in response to hormone stimulation. We find *SYN2* (rank 64 of 22283 (HL60 cells)) rates highest among the genes studied in the experiment, providing strong support for the role of this newly discovered locus in thyroid metabolism. Two other genes, *NRG1* and *CAPZB*, also show evidence of levothyroxine responsiveness in at least one cell line^27^ on the basis of a genome-wide differential expression and rank in the top 5th percentile (Supplementary Table 11). Publicly available data on altered SYN2 expression in brain, limb and tail from control and levothyroxine-treated *Xenopus laevis* during metamorphosis also provide evidence for the relevance of *SYN2* in thyroid function^28^.

## Discussion

In this study, we demonstrate the utility of WGS data (and SNP array data when deeply imputed to WGS reference panels) in appraising the genetic architecture of thyroid function. Using WGS data, we identify a rare functional variant in *TTR* that appears to drive the observed association between an uncommon novel variant near *B4GALT6* and FT4, and we demonstrate a novel association with FT4 arising from rare aggregates in *NRG1*. We also show that common variants collectively account for over 20% of the variance in TSH and FT4, a substantial advance on using only the ‘top SNPs’ from earlier GWA studies^10^. Taken together, this work indicates that both common variants with modest effects and rare variants with larger effects might explain a substantial proportion of the missing heritability of thyroid function, but larger studies are required to identify these variants. Studies including individuals with subclinical thyroid disease, particularly those who are negative for thyroid autoantibodies, may be particularly rewarding, as rare genetic variants with large effect sizes may be associated with serum TSH and FT4 concentrations outside the inclusion ranges we used and therefore would not be detected in our analyses.

Such endeavours are clinically relevant, as there has been a dramatic increase in levothyroxine prescribing for borderline TSH levels^29^. At least three loci identified in this study show evidence of responsiveness to levothyroxine in cell line models, underscoring that borderline TSH levels often reflect the influence of genetic variation rather than overt autoimmune thyroid disease, in which case thyroid hormone replacement may not be appropriate. Our results indicate that further investigation of TSH heterogeneity at the population level is necessary.

## Methods

### Cohorts

Seven populations were used in this study. They are known as the TwinsUK WGS cohort, the TwinsUK GWAS cohort, the ALSPAC WGS cohort, the ALSPAC GWAS cohort, the SardiNIA cohort, the ValBorbera cohort and the Busselton Health Study cohort. Summary statistics of each cohort and full descriptions are given in Supplementary Methods, Supplementary Tables 1 and 2. All human research was approved by the relevant institutional ethics committees.

### WGS data generation

Low-read depth WGS was performed in the TwinsUK and ALSPAC as part of the UK10K project. The SardiNIA cohort also had WGS data available (see Supplementary Methods).

### Statistical analysis

An inverse normal transformation was applied to each trait within each cohort. Age, age^2^, gender and any other cohort-specific variables (Supplementary Table 1) were applied as covariates. Genotype imputation was performed for relevant cohorts using the IMPUTE^30^, MaCH^31^ or Minimac^32^ software packages, with poorly imputed variants excluded. See Supplementary Table 1 for cohort-specific details.

### Single-point association analysis

Association analysis within each cohort was performed using the SNPTEST v2 (ref. 33), GEMMA (genome-wide efficient mixed model association)^34^, EPACTS (efficient and parallelizable association container toolbox) or ProbABEL^35^ software packages. Cohort-specific quality control filters relating to call rate and Hardy–Weinberg equilibrium were applied (Supplementary Table 1). In our analysis, we assessed the change in standardized thyroid measure by allele using a MAF threshold ≥1% and a genome-wide significance threshold of *P*=1.17 × 10^−08^ (ref. 36). Meta-analyses were performed using the GWAMA (genome-wide association meta analysis) software^37^, which was used to perform fixed-effect meta-analyses using estimates of the allelic effect size and s.e. Two meta-analyses were performed for each phenotype: a meta-analysis of the two UK10K WGS cohorts and a meta-analysis of all seven cohorts. The ValBorbera cohort does not have FT4 phenotype data, so this cohort was not included in the meta-analyses for this phenotype. In the meta-analyses, any variants that were missing from >2 cohorts or with a combined MAF ≤1% were excluded. However, in the discovery analyses, a MAF of 0.5% in either cohort was accepted to prevent marginal MAF dropouts; the MAF <1% exclusion was then applied during the meta-analysis.

### Conditional analysis

A conditional analysis was performed to identify independent association signals. Each study re-analysed significant loci using the lead SNP identified in the primary analysis (Table 1) as the conditioning marker. In cohorts where the lead SNP was not available, the best proxy was included (*r*^2^>0.8). A meta-analysis was then performed on these conditional results, using the same methods and filters as described above. The standard genome-wide significant cut-off (*P*<5 × 10^−8^) was used to identify secondary associations.

### Estimation of phenotypic variance explained by genetic variants

We undertook GCTA using WGS data in the ALSPAC and TwinsUK discovery cohorts and data from the SardiNIA and Busselton cohorts to estimate the variance explained by all common SNPs (MAF>1%) in the genome for TSH and FT4, using the GCTA method of Yang *et al*.^12^ We fitted linear mixed-effect models to estimate the phenotypic variance attributable to the common SNPs (*h*_*g*_^*2*^). In these data sets, SNPs were thinned to a set of 2,203,581 approximately independent SNPs using the –indep-pairwise option in PLINK with an LD threshold of *r*^2^>0.2, window size of 5,000 SNPs and step of 1,000 SNPs. A genomic relationship matrix was generated for unrelated individuals, namely, those with genomic correlation <0.025. Estimates were calculated on SNPs filtered for Hardy–Weinberg equilibrium *P* value ≥1 × 10^−6^ and MAF ≥0.01. The genetic and residual variance components were estimated by the restricted maximum likelihood (REML) procedure for different MAF thresholds and for SNPs within a 250 kb window of known markers of thyroid function.

### Expression quantitative trait loci analysis

Data for this study were available from a large-scale genetic association study of human gene expression traits in multiple disease-targeted tissue samples including subcutaneous fat, lymphoblastoid cell lines and whole skin, derived from 856 monozygotic (MZ) and dizygotic (DZ) female twins from the TwinsUK cohort, as part of the MuTHER project^18^. We interrogated only lead SNPs (or proxies in LD, *r*^2^>0.8) using Genevar software^17^. For whole-blood eQTL studies, samples were obtained from a large population-based study^38^. The whole-blood eQTL results were downloaded from the GTex Browser at the Broad Institute on 26 November 2013^39^. We identified alias rsIDs for significant index SNPs using JLIN software and UK10K WGS data. Associations at *P*<1 × 10^−3^ were considered significant.

### DNA methylation analysis

DNA methylation profiles were obtained in whole-blood samples from 279 MZ and DZ twins from the TwinsUK cohort using the Illumina Infinium HumanMethylation450 BeadChip. Illumina beta values were quantile normalized to a standard normal distribution and corrected for chip, order of the sample on the chip, bisulfite-converted DNA concentration and age. The resulting values were used for meQTL analysis, which was performed separately in two samples, first in 149 unrelated individuals from the TwinsUK WGS sample and second in 130 individuals with deeply imputed data from the TwinsUK GWAS sample. MeQTL analysis was performed for each sample in PLINK by fitting an additive model and meta-analysis across the two samples was performed in GWAMA, where we considered results without strong evidence for heterogeneity (Cochran’s Q *P*>0.05 and *I*^2^<0.7). We analysed genotype data at 17 sequence variants (from Table 1), where for each variant meQTL analysis was performed with all DNA methylation array CpG sites located within 50 kb of the variant, resulting in 265 pair-wise tests. MeQTL results (Supplementary Table 8) are presented for variants with nominally significant associations in both the WGS and GWAS samples less than a meta-analysis *P*-value of 1 × 10^−04^. In the *PDE8B* gene, we also considered meQTL effects at the eQTL rs251429 (Supplementary Table 7) and found nominally significant association with DNA methylation at CpG site cg16461538 (*B*=−0.18, s.e.=0.08, *P*=0.02). We assessed the association between DNA methylation levels at the CpG sites identified to harbour meQTLs in our study (Supplementary Table 8) and TSH and FT4 levels. Using the same study design as that adopted in the meQTL analysis, we obtained no nominally significant association between DNA methylation at the 11 CpG sites (Supplementary Table 8) for TSH or FT4 levels. Subsequent replication of meQTL associations observed in TwinsUK was performed in the ALSPAC cohort for which DNA methylation profiles from whole blood were available in 745 individuals. Here, data were rank transformed to follow the normal distribution and then regressed against batch number. Analyses were also performed using PLINK, adjusting for age, sex, top 10 PCs (genetic) and houseman-estimated cell counts (to account for cellular heterogeneity).

### Rare variant analysis

We conducted GWAS candidate gene (AADAT, ABO, B4GALT6, CAPNS2, CAPZB, DIO1, DIRC3, ELK3, FBXO15, FGF7, FOXA2, FOXE1, GLIS3, HACE1, IGFBP2, IGFBP5, INSR, ITPK1, LHX3, LOC440389/LOC102467146, LPCAT2, MAF, MBIP, MIR1179, NETO1, NFIA, NKX2-3, NR3C2, NRG1, PDE10A, PDE8B, PRDM11, RAPGEF5, SASH1, SIVA1, SLC25A52, SOX9, SYN2 TMEM196, TPO, TTR, VAV3, VEGFA)-based analyses to test for association of the combined effects of rare variants on TSH and FT4 using SKAT-O software^40^. This approach maximizes statistical power by applying both burden-based and SKATs. We used the TwinsUK and ALSPAC WGS data to examine loci with a known association with TSH and FT4. We examined all SNPs within the candidate gene regions, including variants within 50 kb on either side of the gene with MAF <1% down to a MAF of 0.04% (in a cohort), or 0.02% (overall). These analyses used sequential non-overlapping windows each containing 50 SNPs. Association at *P*<1.55 × 10^−5^ (Bonferroni corrected) was considered significant. For the meta-analysis of rare variant data from the WGS cohorts, we used SkatMeta^41^.

### Polygenic score analysis

We conducted polygenic score analyses to test for substantive polygenic effects on TSH and FT4 and for a shared polygenic basis between thyroid traits and a range of related phenotypes including key cardiovascular traits, metabolic, anthropometric, endocrine and bone traits. Polygenic scores have been used to summarize genetic effects for an ensemble of markers that may not individually achieve significance but are relevant to regulation of the trait. The composite score represents an overall genetic signal and can then be used to obtain evidence of a common genetic basis for related disorders^42^. We ranked SNPs by their marginal association with TSH and FT4 using the meta-analysis data set, with TwinsUK samples excluded (leaving *N*=13,874 for TSH and *N*=12,561 for FT4). SNPs were thinned to a set of 2,203,581 approximately independent SNPs using the –indep-pairwise option in PLINK with an LD threshold of *r*^2^>0.2, window size of 5,000 SNPs and step of 1,000 SNPs. On the basis of their associations in the meta-analysis data, SNPs were selected for constructing polygenic scores according to a range of *P* value thresholds. Scores were then constructed for subjects in the TwinsUK data sets by forming the weighted sum of trait-increasing alleles, with the weights taken as the effect size in the meta-analysis data. To construct polygenic scores, we used 67 SNPs (rs10028213, rs10030849, rs10032216, rs10420008, rs10499559, rs10519227, rs10799824, rs10917469, rs10917477, rs11103377, rs113107469, rs11624776, rs116552240, rs116909374, rs11694732, rs11726248, rs11755845, rs12410532, rs13015993, rs1537424, rs1571583, rs17020124, rs17723470, rs17776563, rs2046045, rs2235544, rs2396084, rs2439302, rs28435578, rs2928167, rs3008034, rs3008043, rs310763, rs334699, rs334725, rs34269820, rs3813582, rs4704397, rs4804416, rs56738967, rs6082762, rs61938844, rs6499766, rs6885099, rs6923866, rs6977660, rs7128207, rs7190187, rs7240777, rs729761, rs73362602, rs73398284, rs737308, rs753760, rs7568039, rs7694879, rs7825175, rs7860634, rs7864322, rs7913135, rs9322817, rs944289, rs9472138, rs9497965, rs965513, rs966423 and rs9915657) that have been shown to be associated with thyroid hormone levels^8^,^10^,^26^. The polygenic score was then tested for association with relevant thyroid and other phenotypes in the TwinsUK sample.

### Chemogenomic analysis

To identify putative thyroxine-responsive genes among the candidate loci (*AADAT*, *ABO*, *B4GALT6*, *CAPZB*, *DIO1*, *FOXE1*, *IGFBP2*, *LHX3*, *MAF*, *MBIP*, *MFAP3L*, *NR3C2*, *NRG1*, *PDE10A*, *PDE8B*, *QSOX2*, *SLC25A52*, *SYN2*, *TTR* and *VEGFA*), gene expression data measured in response to levothyroxine treatment in a range of cell lines were retrieved from the Connectivity Map resource^27^. We considered a genome-wide differential expression rank in the top 5th percentile among 22,283 probes as evidence of differential expression.

## Author contributions

Cohort collection was done by P.N.T., E.P., G.A., C.M.D., S.N., J.P.B., J.H., E.M.L., V.P., W.W., D.T., J.P.W., C.M.D., T.D.S., G.D.S., R.D., J.B.R., S.S., N.S., N.J.T. and S.G.W. Phenotype cleaning was done by P.N.T., E.P., S.C., P.J.C., M.T., S.J.B., B.H.M., S.S., N.S., N.J.T. and S.G.W. Genotype data processing and cleaning was done by S.J.B., J.M., K.W., Y.M., J.P.B., J.H., S.M., D.M., D.S. and E.Z. Genotype–phenotype association testing was done by P.N.T., E.P., S.C., P.J.C., M.T., S.J.B., B.H.M., H.A.S., M.R.B., P.C., P.D., F.D., V.F., C.G., E.G., A.D.J., J.H., V.P., J.R.B., J.T.B., W.Y., C.R., T.G., G.L.S. and H.-F.Z. Bioinformatics by S.C., P.J.C., B.H.M., S.J.B., J.M., K.W., Y.M., S.G.W., J.R.B.P., M.R.B., P.D. and F.D. Manuscript drafting was done by P.N.T., E.P., S.C., P.J.C., M.T., S.J.B., B.H.M., J.P.W., C.M.D., J.P.W., J.B.T., M.R.B., J.R.B.P., F.D., S.S., N.J.T. and S.G.W. All authors critically revised the manuscript.

## Additional information

**How to cite this article**: Taylor, P. N. *et al*. Whole-genome sequence-based analysis of thyroid function. *Nat. Commun.* 6:5681 doi: 10.1038/ncomms6681 (2015).

## Supplementary Material

Supplementary Information Supplementary Figures 1-6, Supplementary Tables 1-11, Supplementary Methods and Supplementary References

## Figures and Tables

**Table 1:** Independent SNPs with MAF≥1% associated with serum TSH and FT4 levels in the overall meta-analysis.

**Gene**	**SNP**	**Chromosome**	**Position**	**A1/A2**	**Freq A1**	**Effect**	**Std Err**	***N***	***P***	**Het** ***P***
TSH										
CAPZB	rs12410532	1	19845279	T/C	0.164	−0.090	0.016	16,332	9.41 × 10^−9^	0.003
IGFBP2	rs7568039	2	217612321	A/C	0.250	−0.122	0.014	16,335	2.11 × 10^−19^	0.370
**SYN2**	**rs310763**	**3**	**12230704**	**T/C**	**0.235**	**0.083**	**0.014**	**16,334**	**6.15 × 10**^−**9**^	**0.252**
NR3C2	rs28435578	4	149646538	C/T	0.227	−0.166	0.014	16,333	4.59 × 10^−32^	0.109
PDE8B	rs2046045	5	76535811	G/T	0.414	0.142	0.012	16,334	4.05 × 10^−33^	0.653
**PDE8B**	**rs2928167**	**5**	**76477820**	**G/A**	**0.104**	−**0.145**	**0.019**	**16334**	**5.94 × 10**^−**14**^	**0.994**
VEGFA	rs6923866	6	43901184	C/T	0.280	−0.102	0.013	16,333	7.55 × 10^−15^	0.646
VEGFA	rs2396084	6	43804825	A/G	0.287	−0.096	0.013	16,333	4.33 × 10^−13^	0.422
PDE10A	rs3008034	6	166043862	C/T	0.312	−0.131	0.012	16,335	4.68 × 10^−26^	0.084
FOXE1	rs112817873	9	100548934	T/A	0.323	−0.140	0.015	11,544	6.15 × 10^−20^	2.02 × 10^−6^
ABO	rs116552240	9	136149098	A/T	0.239	0.121	0.016	14,047	1.92 × 10^−14^	4.11 × 10^−4^
MBIP	rs116909374	14	36738361	T/C	0.043	−0.208	0.032	15,037	4.69 × 10^−11^	0.179
MAF	rs17767742	16	79740541	G/C	0.354	−0.113	0.012	16,335	5.64 × 10^−20^	0.447
										
FT4										
DIO1	rs2235544	1	54375570	A/C	0.499	0.154	0.013	13,650	5.23 × 10^−34^	0.084
AADAT	rs7694879	4	170969799	T/C	0.095	0.137	0.022	13,650	4.15 × 10^−10^	0.168
LHX3	rs11103377	9	139097135	G/A	0.496	0.087	0.013	13,651	1.44 × 10^−11^	0.735
**B4GALT6**	**rs113107469**	**18**	**29306737**	**T/C**	**0.032**	**0.223**	**0.037**	**13,649**	**1.27 × 10**^−**9**^	**0.574**

FT4, free thyroxine; MAF, minor allele frequency; SNP, single nucleotide polymorphism; TSH, thyrotropin.

Table shows the association results for SNPs that reached genome-wide level significance in the final meta-analysis. For each SNP, the best candidate gene is showed, as well as its genomic position, the effect allele (A1), the other allele (A2), the combined frequency of A1 across studies (Freq A1) the effect size (beta—change in standardized thyroid measure by allele) and its standard error (Std Err), the *P* value for association (*P*), the number of samples analysed (*N*) and the *P* values for heterogeneity of effects across the cohorts used in the meta-analysis (Het *P*). Entries in bold reflect novel identified SNPs.
