# Supplementary material for: Whole-genome sequence-based analysis of thyroid function
Source: Nat Commun. 2015 Mar 6;6:5681. doi: 10.1038/ncomms6681 (PMC4366514; doi:10.1038/ncomms6681)
Supplement: Supplementary Information — Supplementary Figures 1-6, Supplementary Tables 1-11, Supplementary Methods and Supplementary References [file ncomms6681-s1.pdf]

Supplementary Figures

Supplementary Figure 1A. QQ plots for TSH and FT4 in the discovery analysis

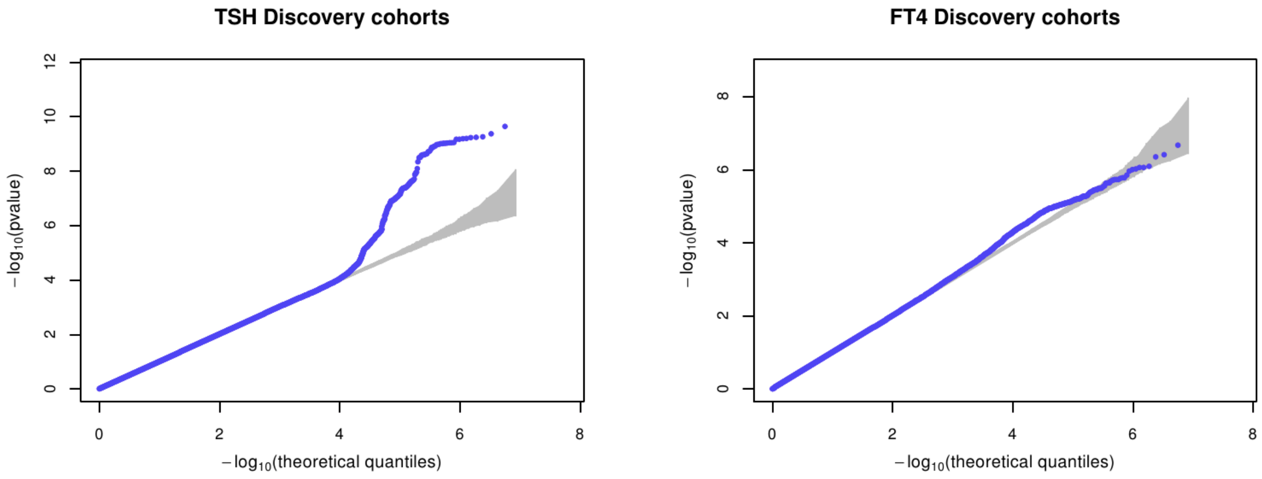

Supplementary Figure 1B. QQ plots for TSH and FT4 in the overall analysis

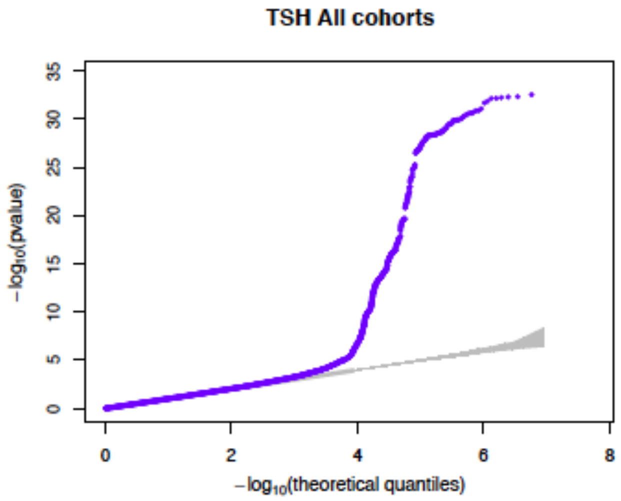

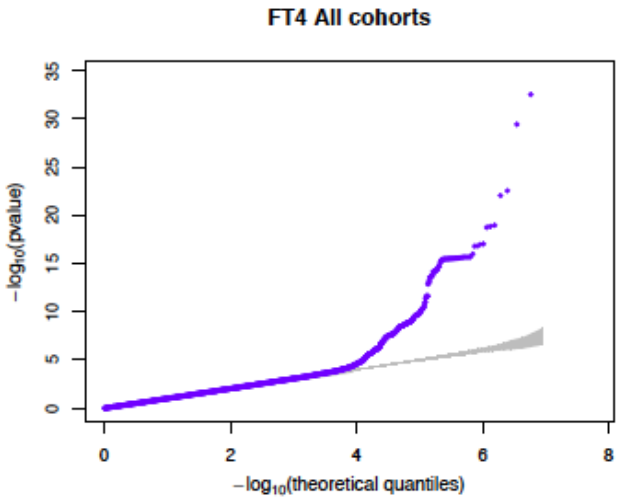

Supplementary Figure 2. Annotated Manhattan plot for TSH in the discovery analysis

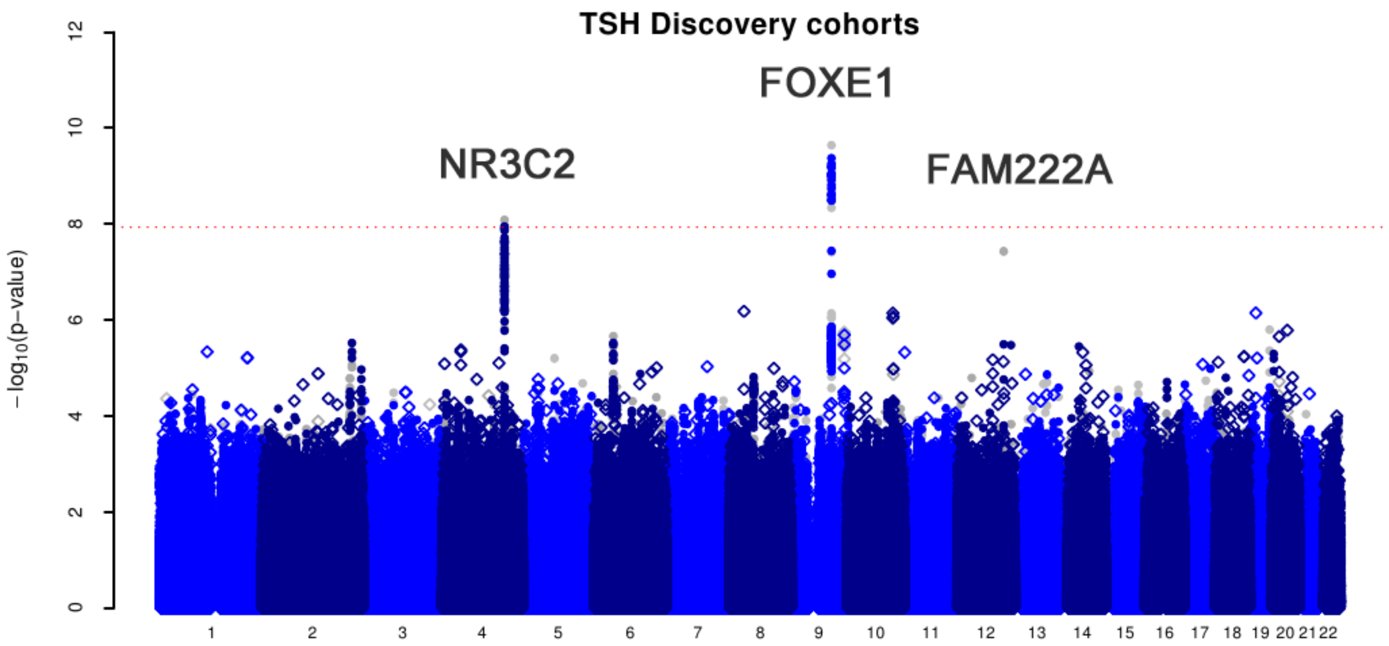

Supplementary Figure 2. Annotated Manhattan plot from the discovery analysis for serum TSH levels. SNPs (MAF>1%) are plotted on the X axis according to their position on each chromosome against association with TSH on the Y axis (shown as–log10 P value). The loci are regarded as genome-wide significant at $P<5x10^{-8}$. Variants with 1%<MAF <5% are show as open diamond symbols. Common SNPs (MAF>5%) are shown as sold circles with those present in Hapmap II reference panels in grey and those derived from WGS or deeply imputed using WGS and 1000 genomes reference panels in blue. SNPs shown as a red asterisk represent novel genome-wide significant ($P<1.17x10^{-8}$) findings.

Supplementary Figure 3. Annotated Manhattan plot for FT4 in the discovery and overall analysis

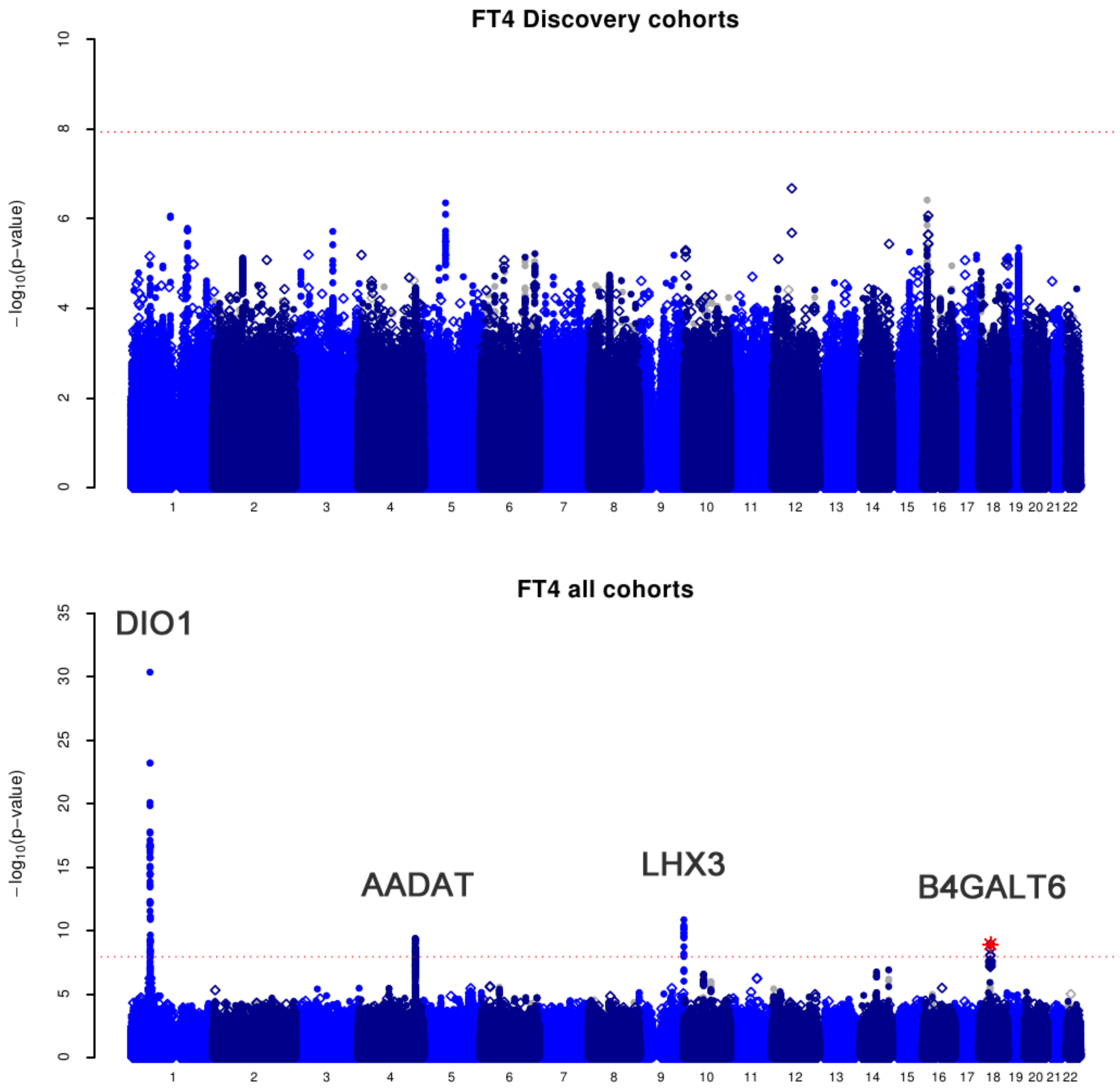

Supplementary Figure 3. Annotated Manhattan plot from the discovery an) overall analysis for serum FT4 levels. SNPs (MAF>1%) are plotted on the X axis according to their position on each chromosome against association with FT4 on the Y axis (shown as–log10 P value). The loci are regarded as genome-wide significant at $P<5x10^{-8}$. Variants with 1%<MAF <5% are show as open diamond symbols. Common SNPs (MAF>5%) are shown as sold circles with those present in Hapmap II reference panels in grey and those derived from WGS or deeply imputed using WGS and 1000 genomes reference panels in blue. SNPs shown as a red asterisk represent novel genome-wide significant ($P<1.17x10^{-8}$) findings.

Supplementary Figure 4A Forest plot of the tops SNP in Table 1 in *CAPZB, IGFBP2* and *SYN2* gene region associated with TSH in each cohort. Squares represent the estimated per-allele beta-estimate for individual studies, error bars are 95%CI. VBI = ValBorbera.

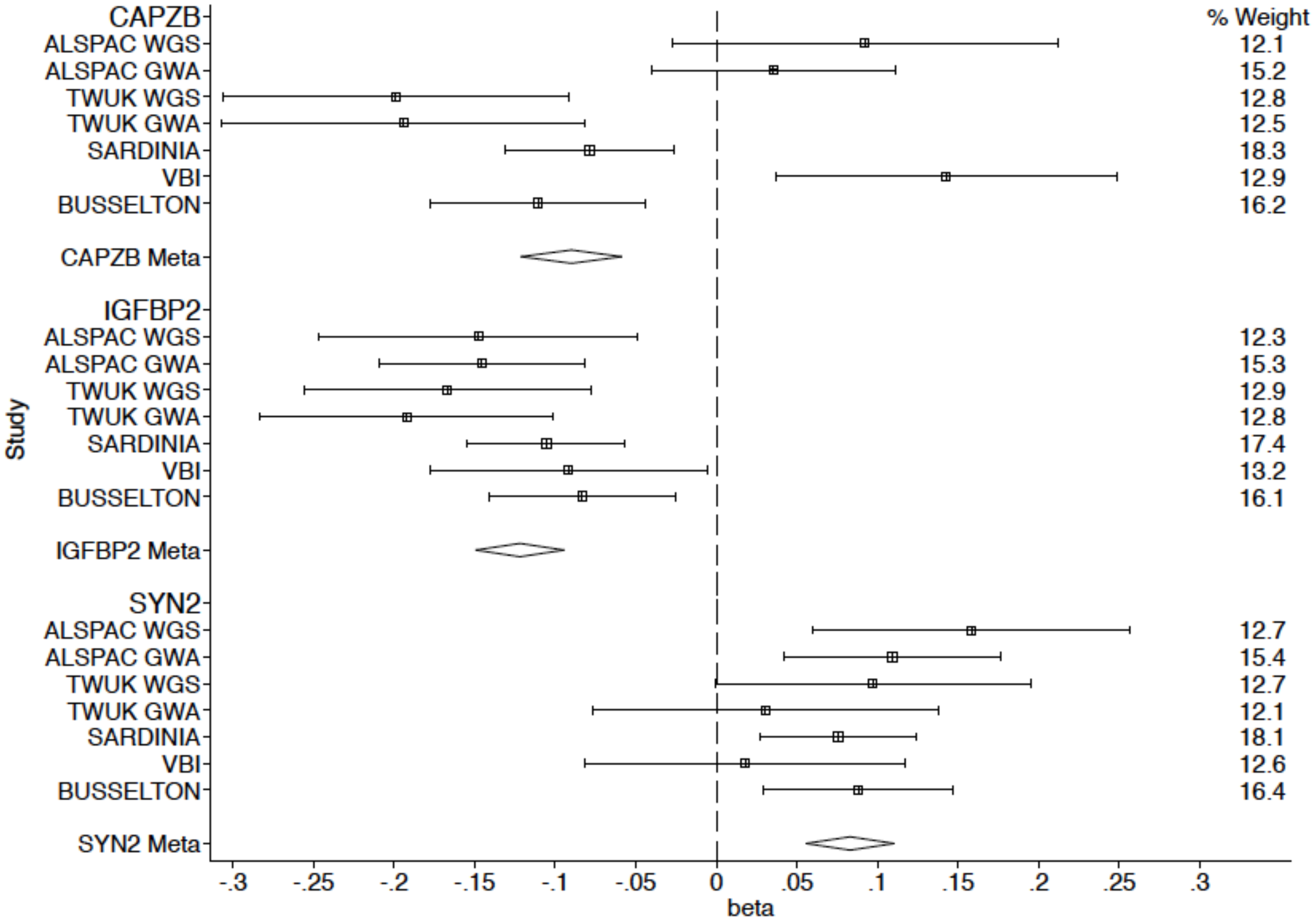

Supplementary Figure 4B Forest plot of the tops SNP in Table 1 in *NR3C2* and *PDE8B* gene region associated with TSH in each cohort. Squares represent the estimated per-allele beta-estimate for individual studies error bars are 95%CI.

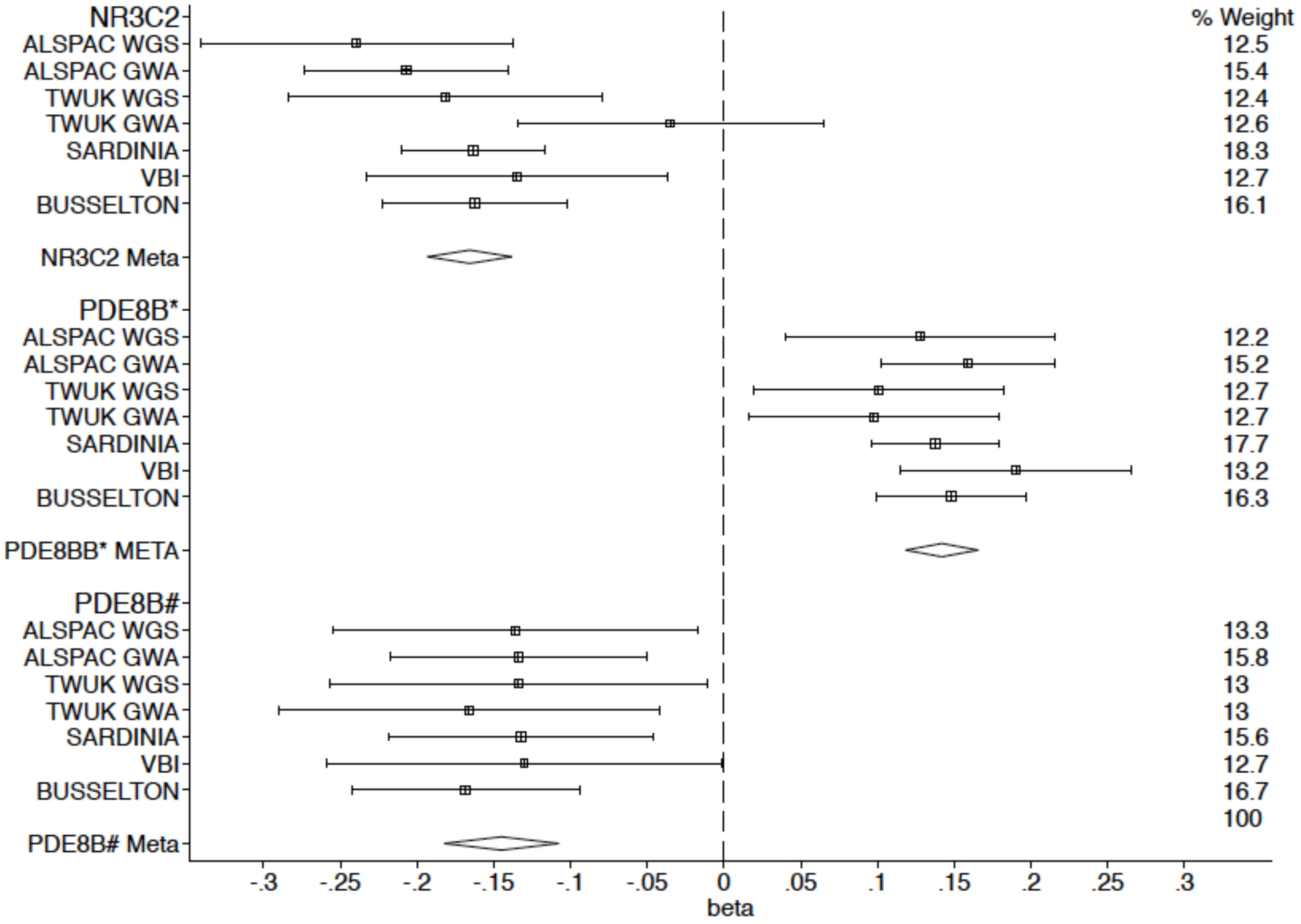

Supplementary Figure 4C Forest plot of the tops SNP in Table 1 in *VEGFA* and *PDE10A* gene region associated with TSH in each cohort. Squares represent the estimated per-allele beta-estimate for individual studies error bars are 95%CI.

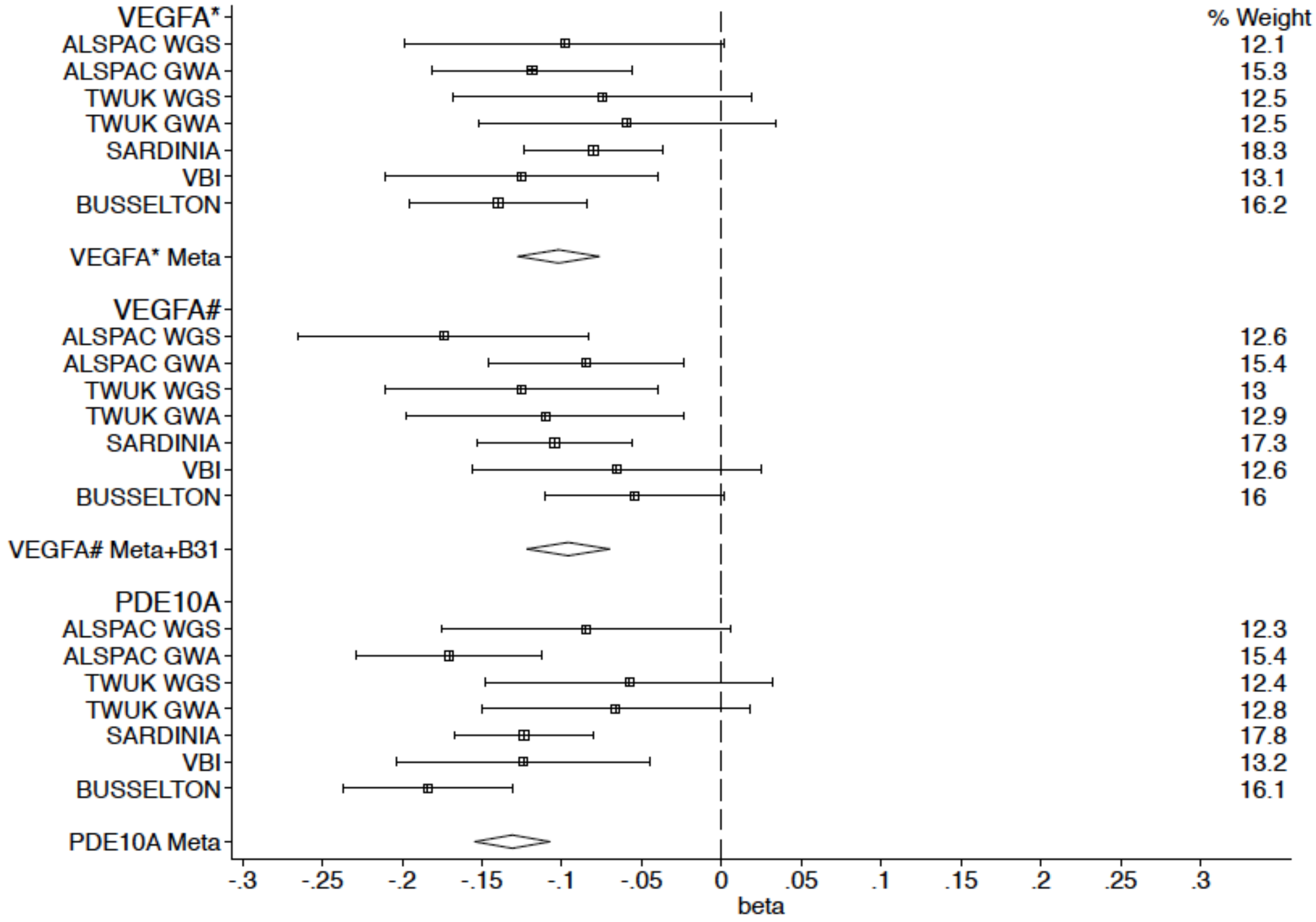

Supplementary Figure 4D Forest plot of the tops SNP in Table 1 in *FOXE1, ABO, MBIP* and *MAF* gene region associated with TSH in each cohort. Squares represent the estimated per-allele beta-estimate for individual studies error bars are 95%CI.

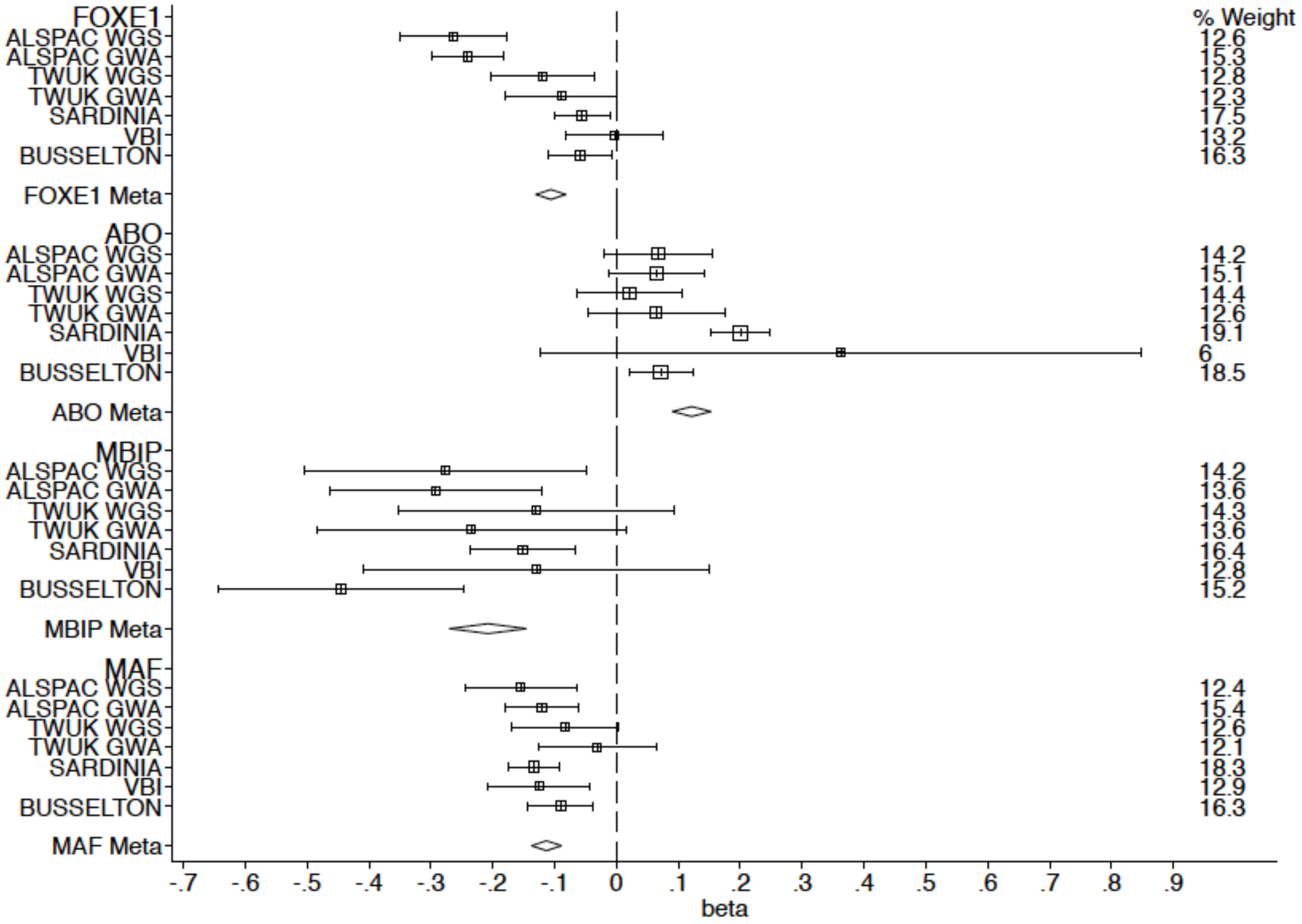

Supplementary Figure 4E Forest plot of the tops SNP in Table 1 in *DIO1, AADAT, LHX3* and *B4GALT6* gene region associated with FT4 in each cohort. Squares represent the estimated per-allele beta-estimate for individual studies error bars are 95%CI.

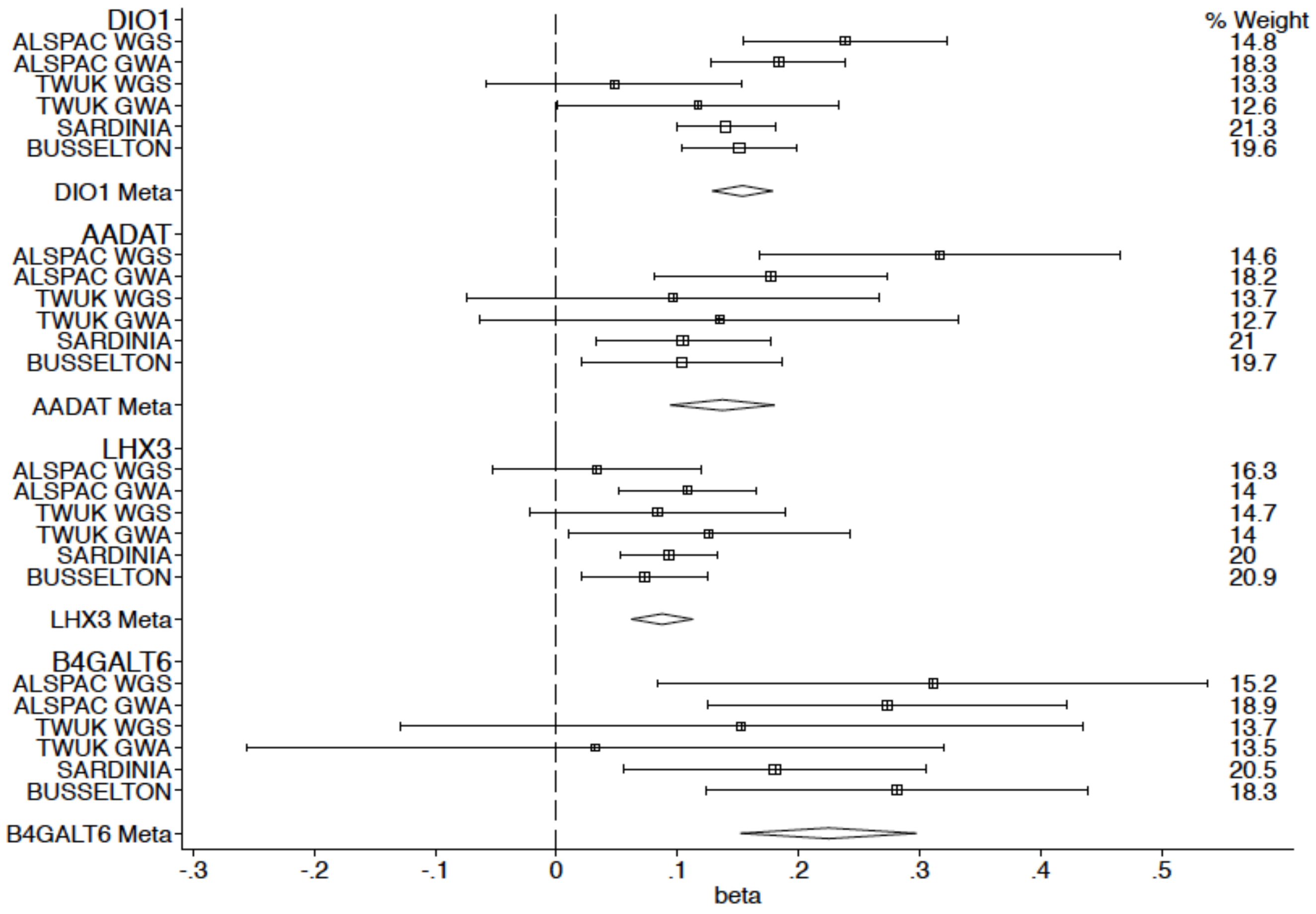

Supplementary Figure 5. Regional association plots showing genome-wide significant loci for serum TSH in the overall meta-analysis.

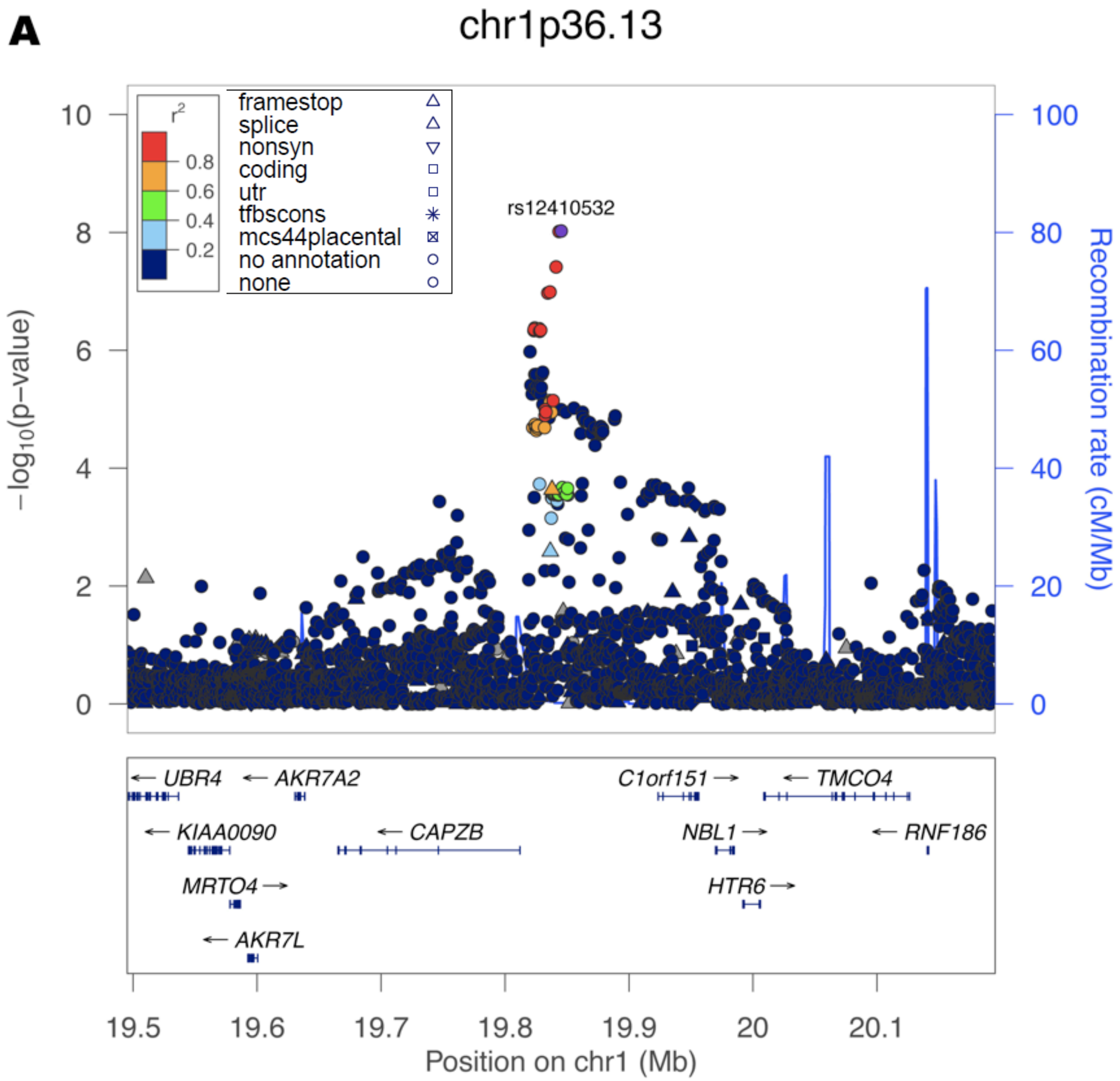

B

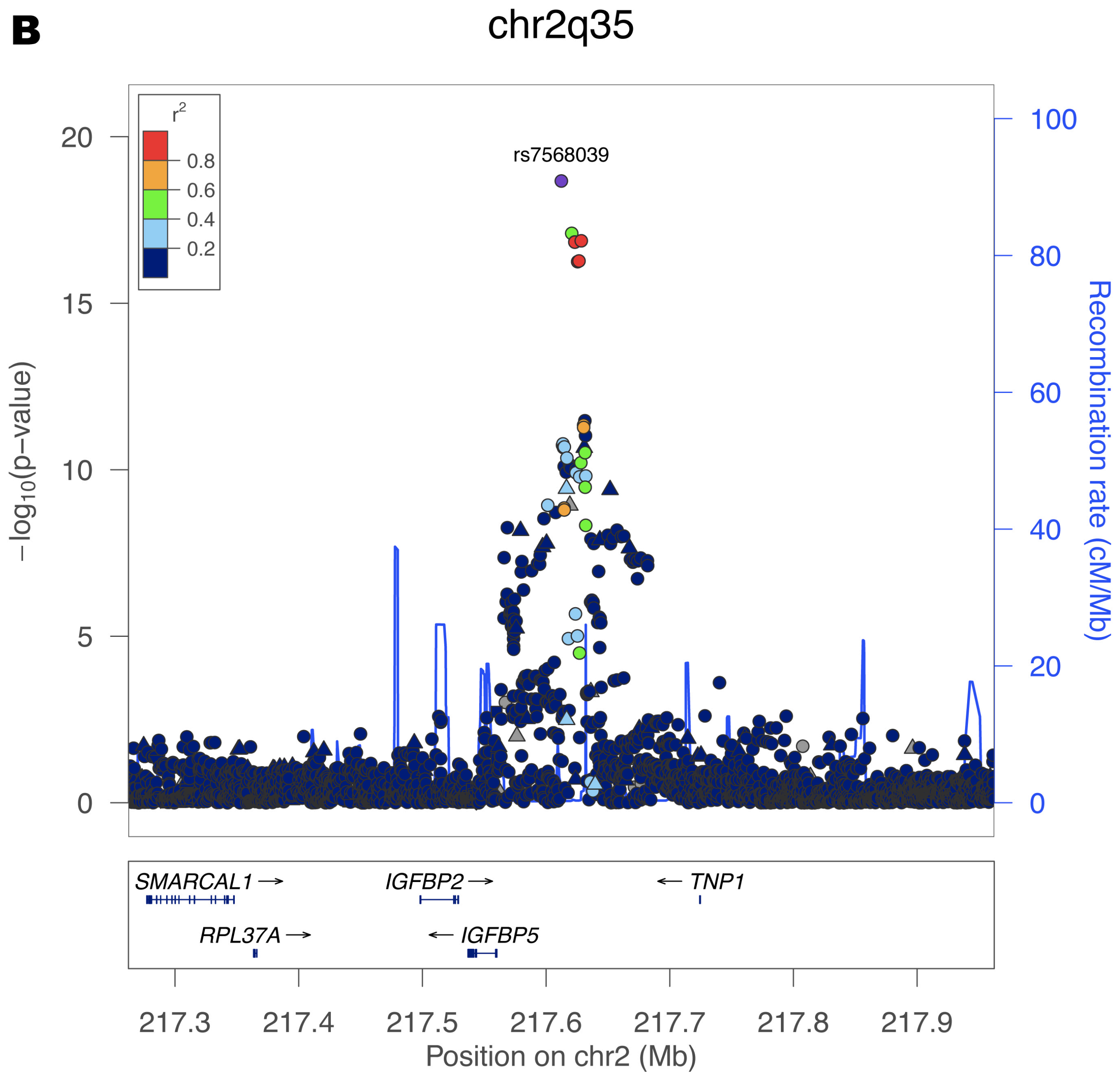
chr2q35
rs7568039
r2
0.8
0.6
0.4
0.2
20
15
10
5
0
−log10(p−value)
100
80
60
40
20
0
Recombination rate (cM/Mb)
SMARCAL1 →
IGFBP2 →
← TNP1
RPL37A →
← IGFBP5
217.3
217.4
217.5
217.6
217.7
217.8
217.9
Position on chr2 (Mb)

c

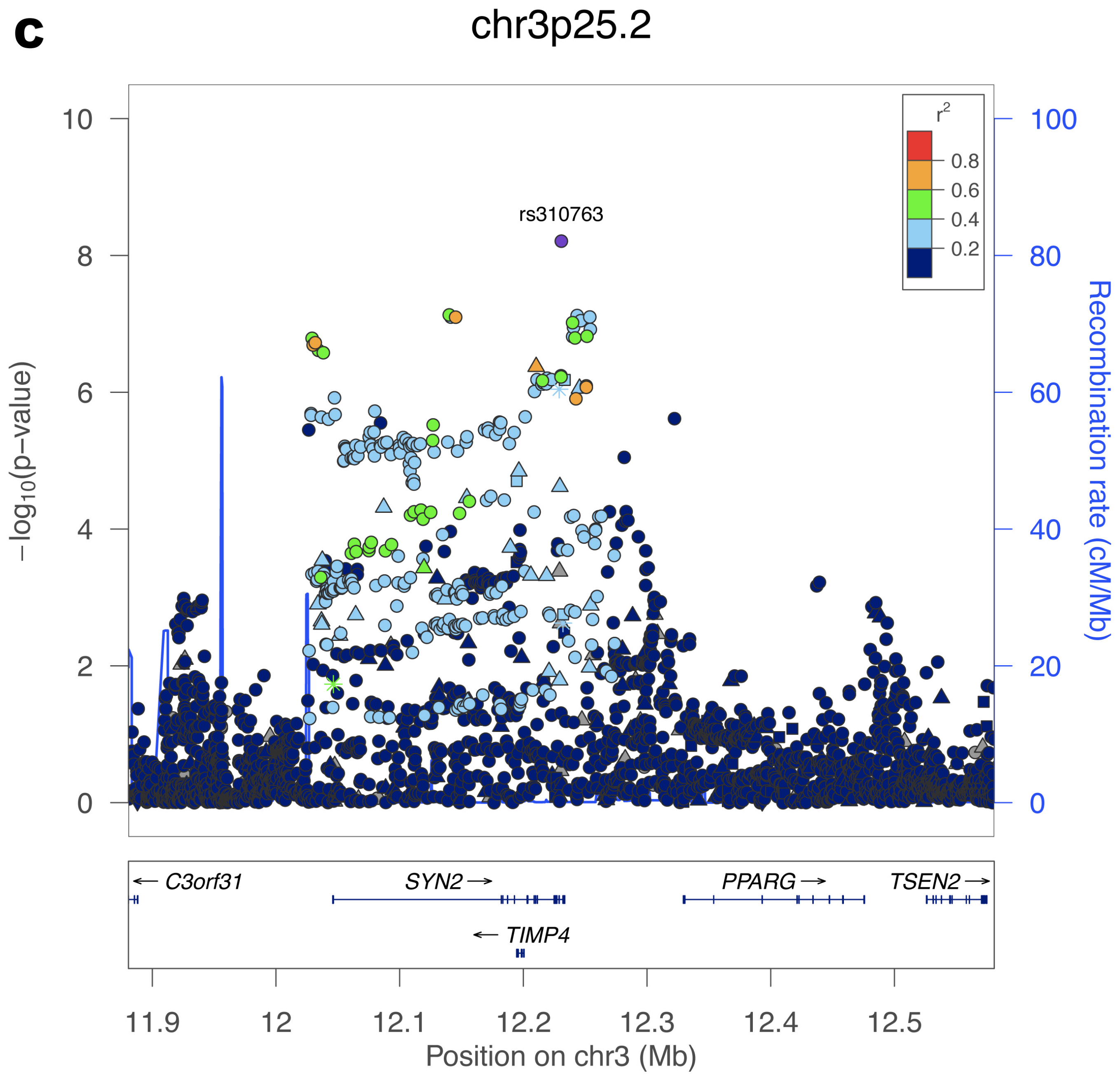
chr3p25.2
rs310763
r2
0.8
0.6
0.4
0.2
10
8
6
4
2
0
100
80
60
40
20
0
−log10(p−value)
Recombination rate (cM/Mb)
← C3orf31
SYN2 →
PPARG →
TSEN2 →
← TIMP4
11.9
12
12.1
12.2
12.3
12.4
12.5
Position on chr3 (Mb)

D

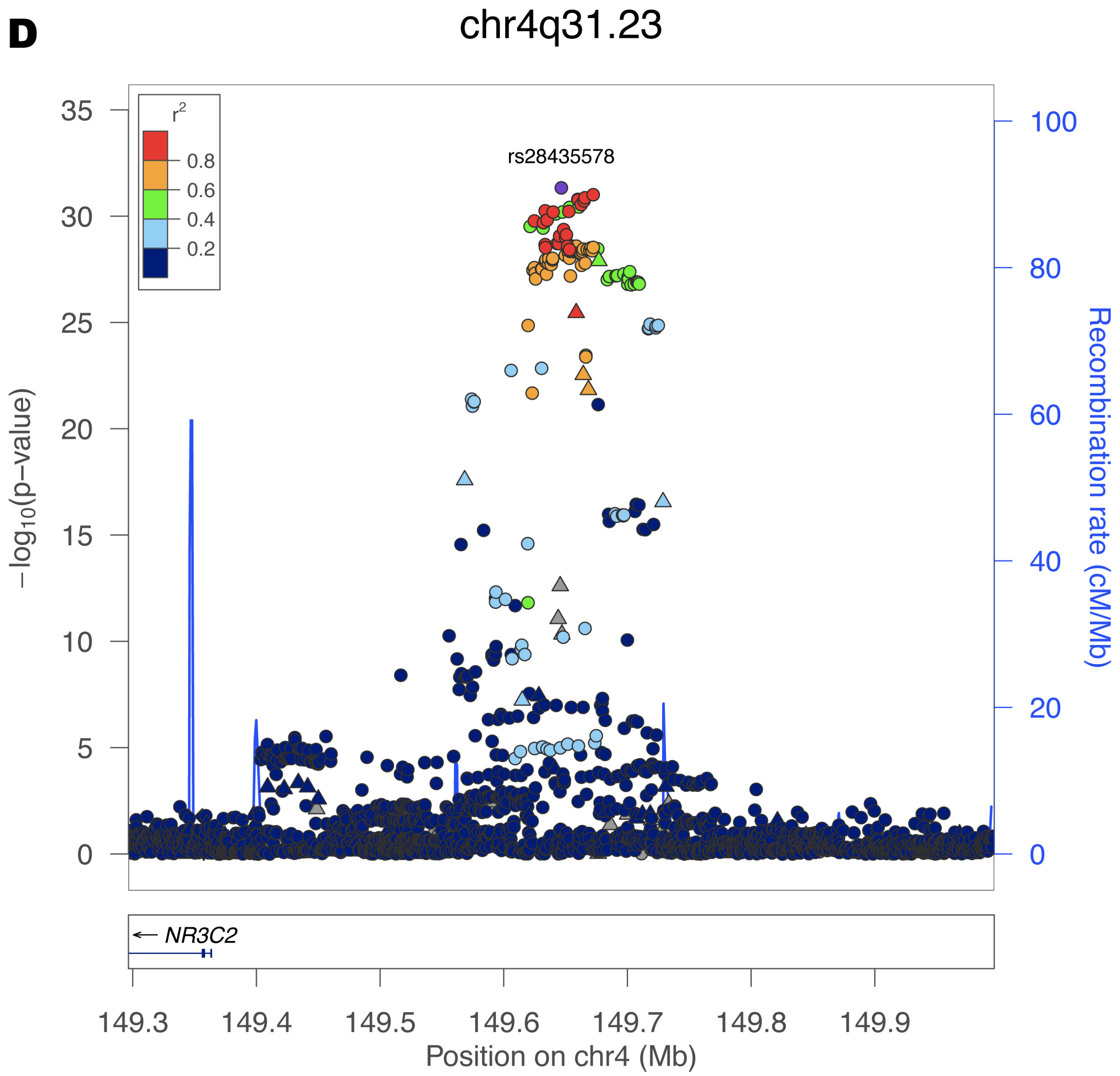
chr4q31.23
rs28435578
r²
0.8
0.6
0.4
0.2
35
30
25
20
15
10
5
0
−log10(p−value)
100
80
60
40
20
0
Recombination rate (cM/Mb)
NR3C2
149.3
149.4
149.5
149.6
149.7
149.8
149.9
Position on chr4 (Mb)

E

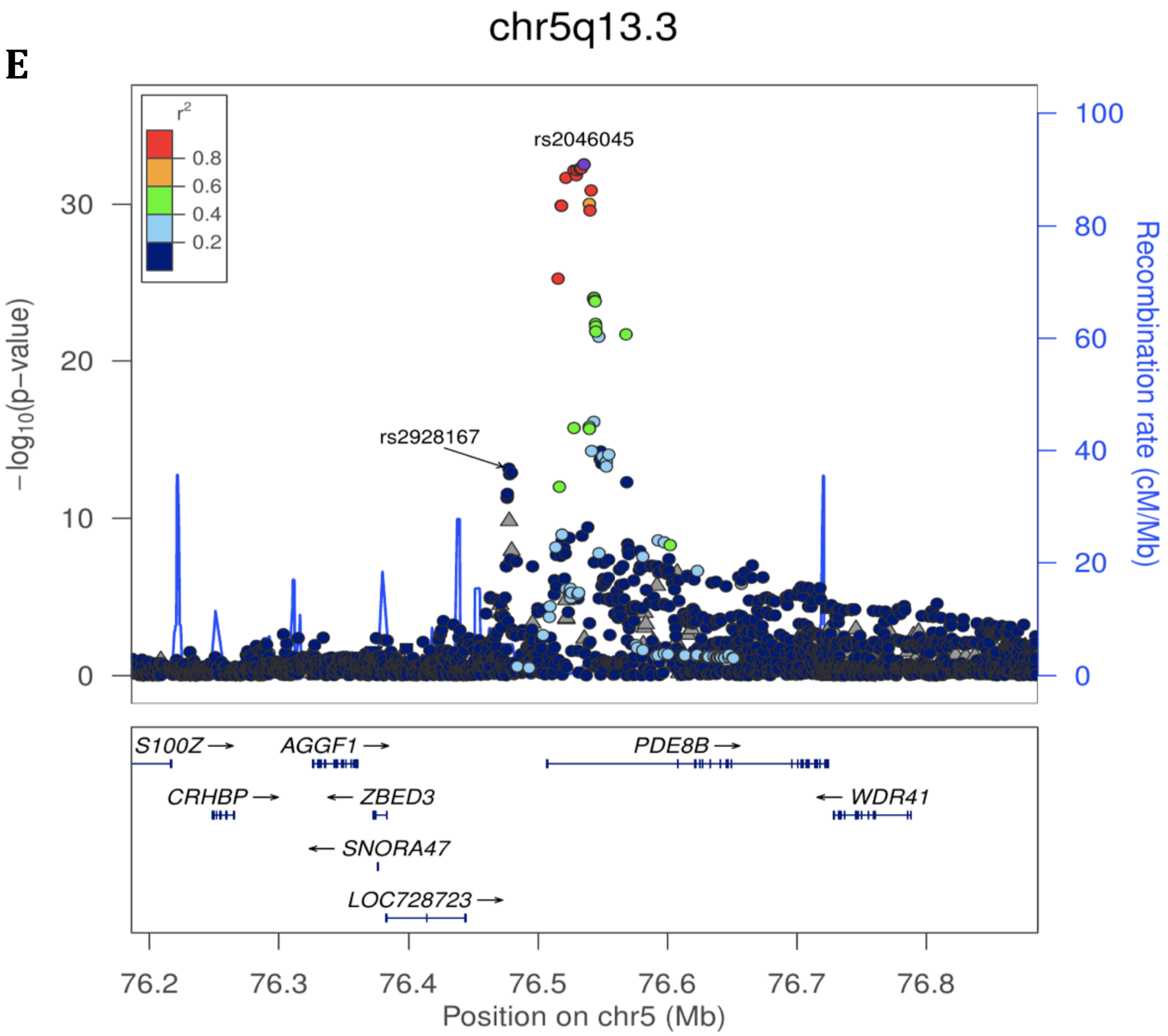
chr5q13.3
r²
0.8
0.6
0.4
0.2
rs2046045
rs2928167
−log10(p−value)
Recombination rate (cM/Mb)
S100Z →
AGGF1 →
PDE8B →
CRHBP →
← ZBED3
← WDR41
← SNORA47
LOC728723 →
76.2
76.3
76.4
76.5
76.6
76.7
76.8
Position on chr5 (Mb)

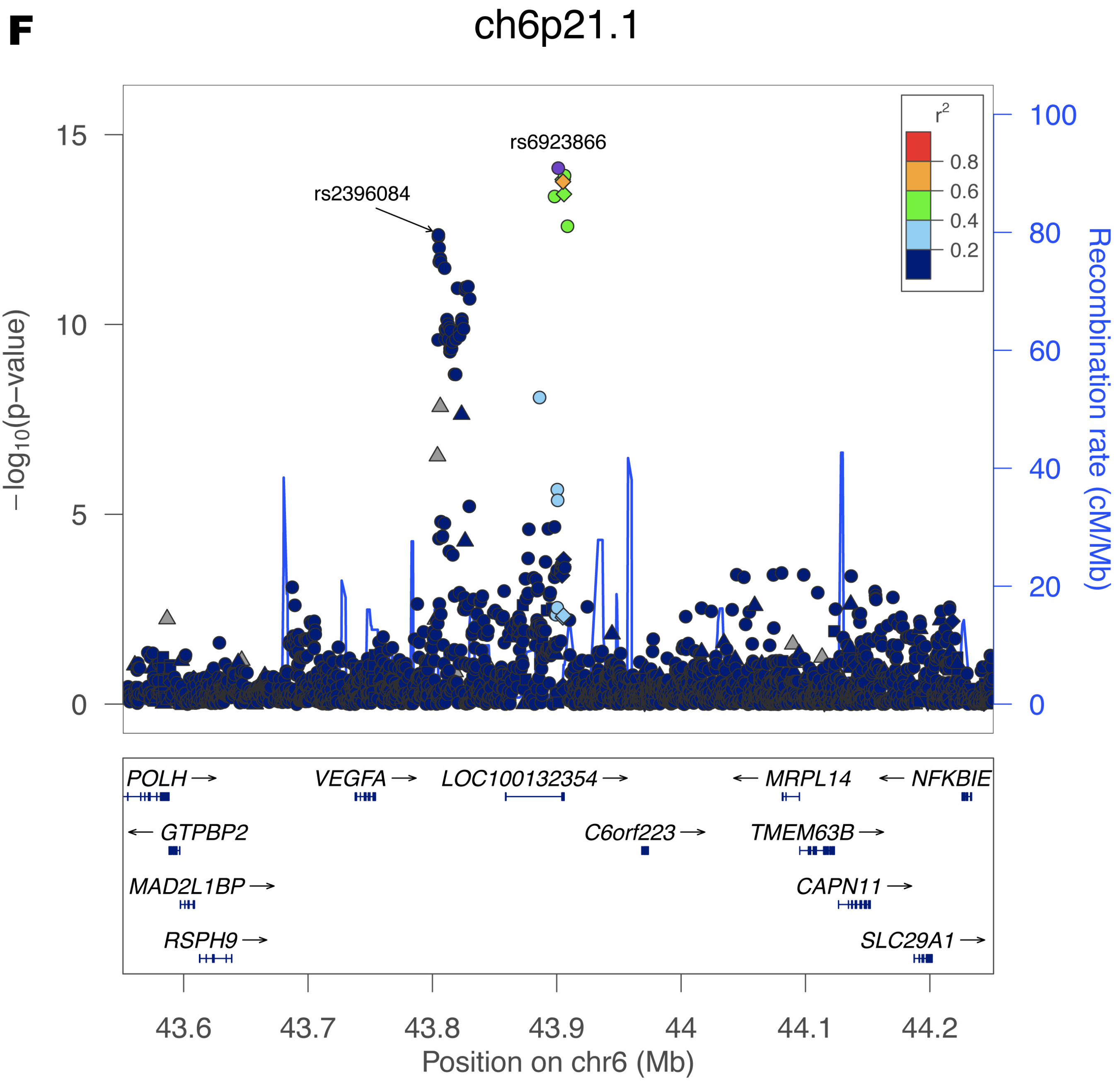
F
ch6p21.1
rs6923866
rs2396084
r2
0.8
0.6
0.4
0.2
−log10(p−value)
Recombination rate (cM/Mb)
POLH
VEGFA
LOC100132354
MRPL14
NFKBIE
GTPBP2
C6orf223
TMEM63B
MAD2L1BP
CAPN11
RSPH9
SLC29A1
Position on chr6 (Mb)

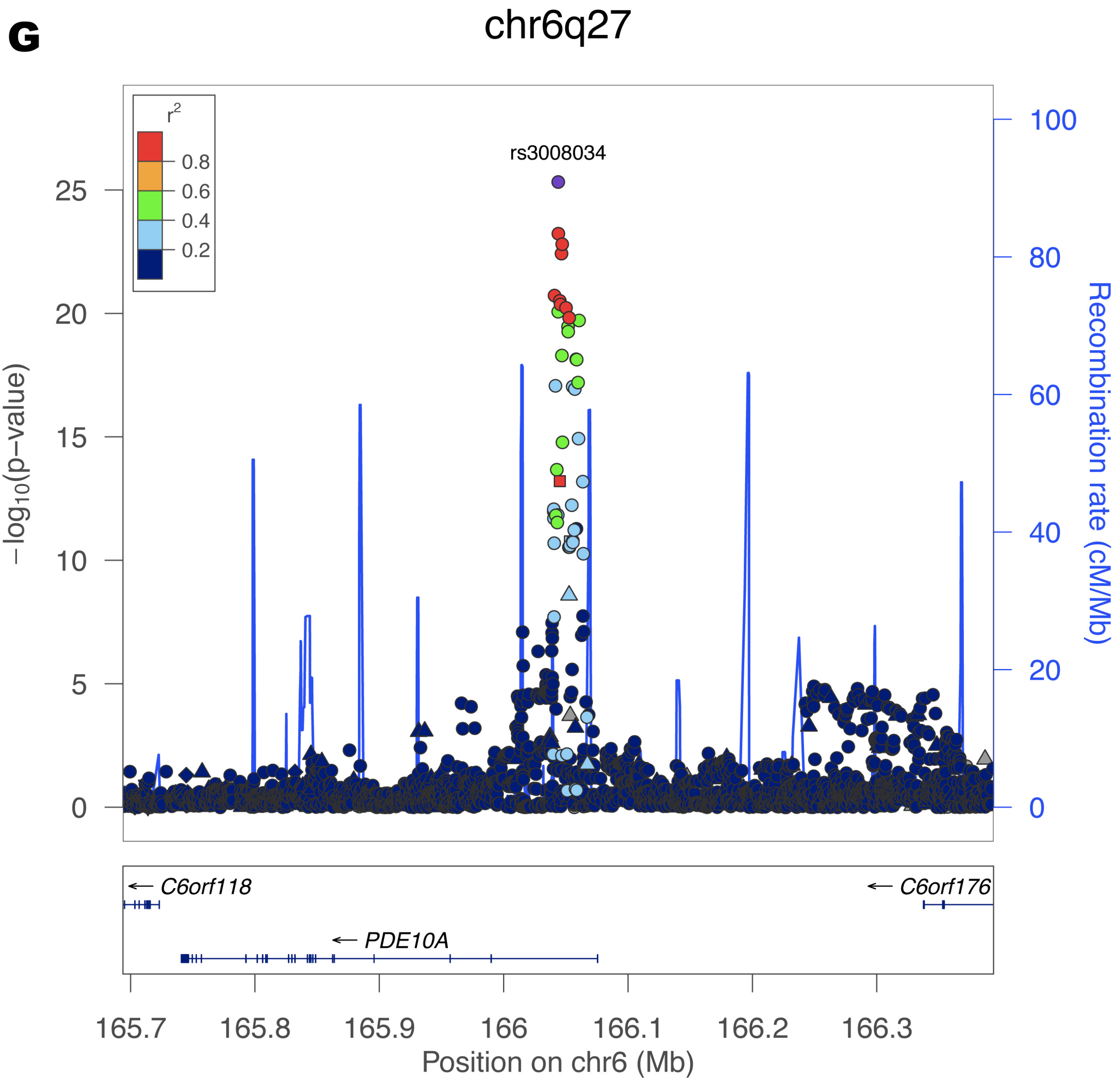
G
chr6q27
rs3008034
r2
0.8
0.6
0.4
0.2
−log10(p−value)
Recombination rate (cM/Mb)
C6orf118
C6orf176
PDE10A
165.7
165.8
165.9
166
166.1
166.2
166.3
Position on chr6 (Mb)

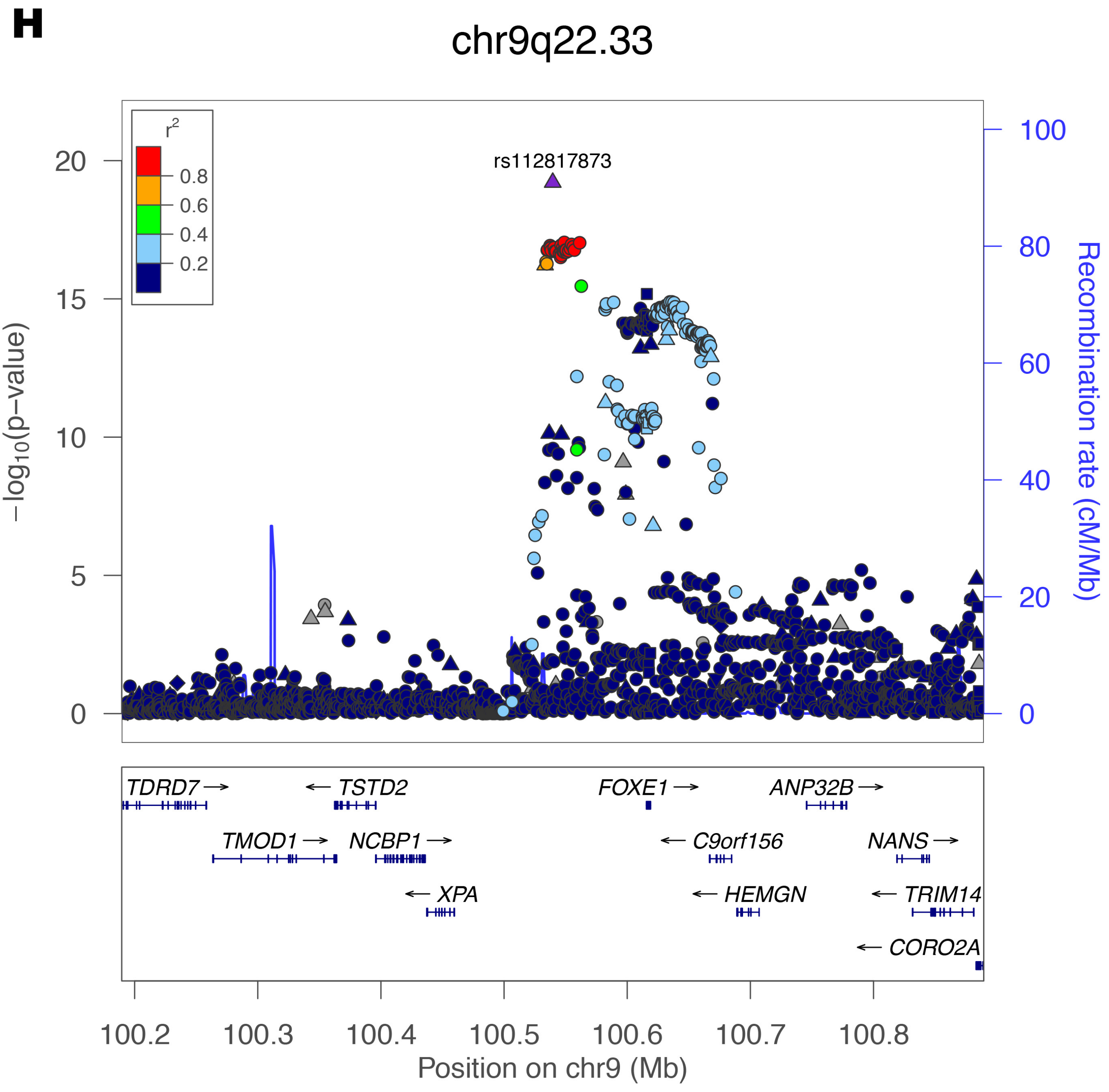
H
chr9q22.33
rs112817873
r2
0.8
0.6
0.4
0.2
−log10(p−value)
Recombination rate (cM/Mb)
TDRD7
TSTD2
FOXE1
ANP32B
TMOD1
NCBP1
C9orf156
NANS
XPA
HEMGN
TRIM14
CORO2A
Position on chr9 (Mb)

I

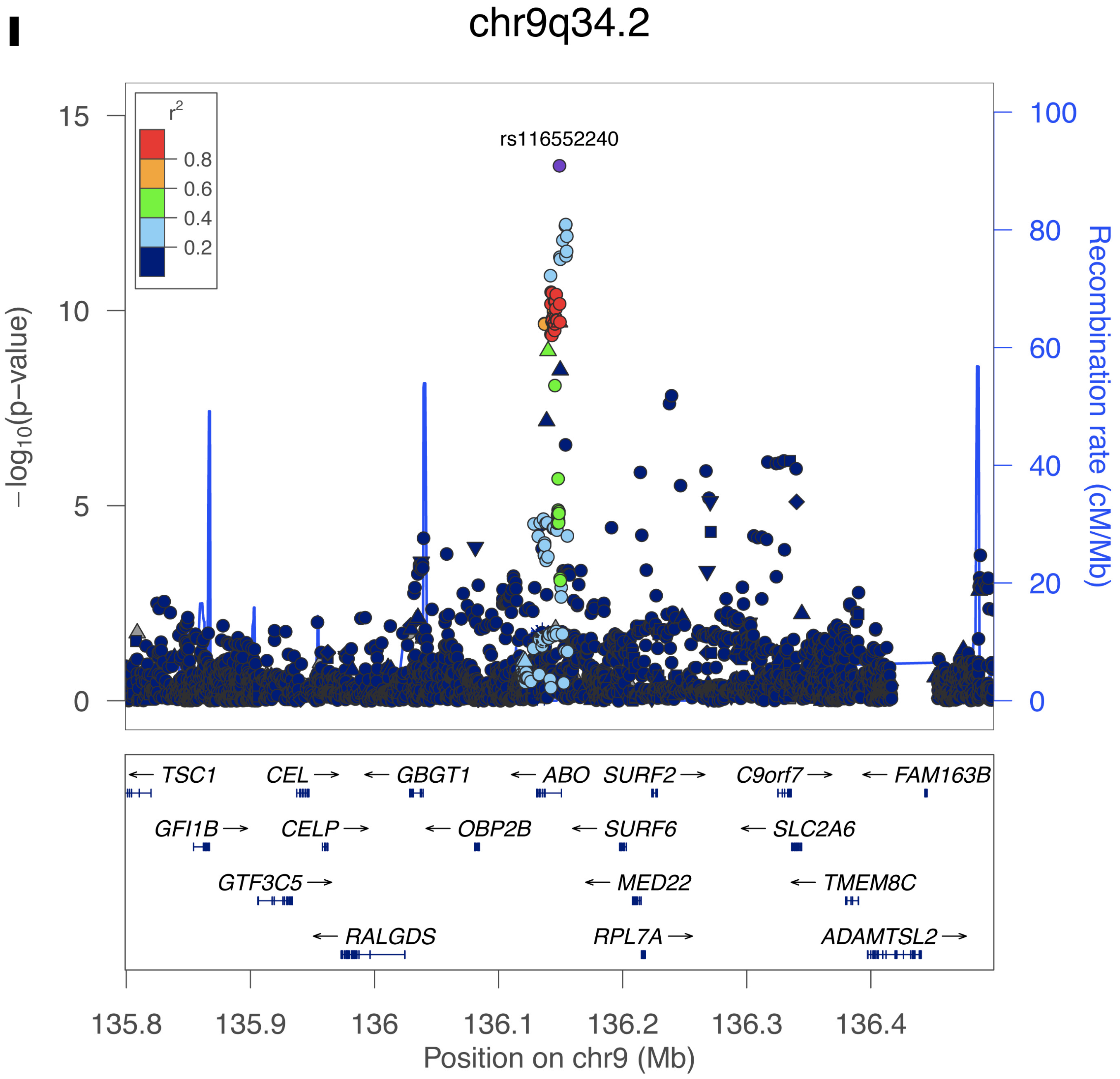

chr9q34.2
rs116552240
r2
0.8
0.6
0.4
0.2
−log10(p−value)
Recombination rate (cM/Mb)
TSC1
CEL
GBGT1
ABO
SURF2
C9orf7
FAM163B
GFI1B
CELP
OBP2B
SURF6
SLC2A6
GTF3C5
MED22
TMEM8C
RALGDS
RPL7A
ADAMTSL2
135.8
135.9
136
136.1
136.2
136.3
136.4
Position on chr9 (Mb)

J

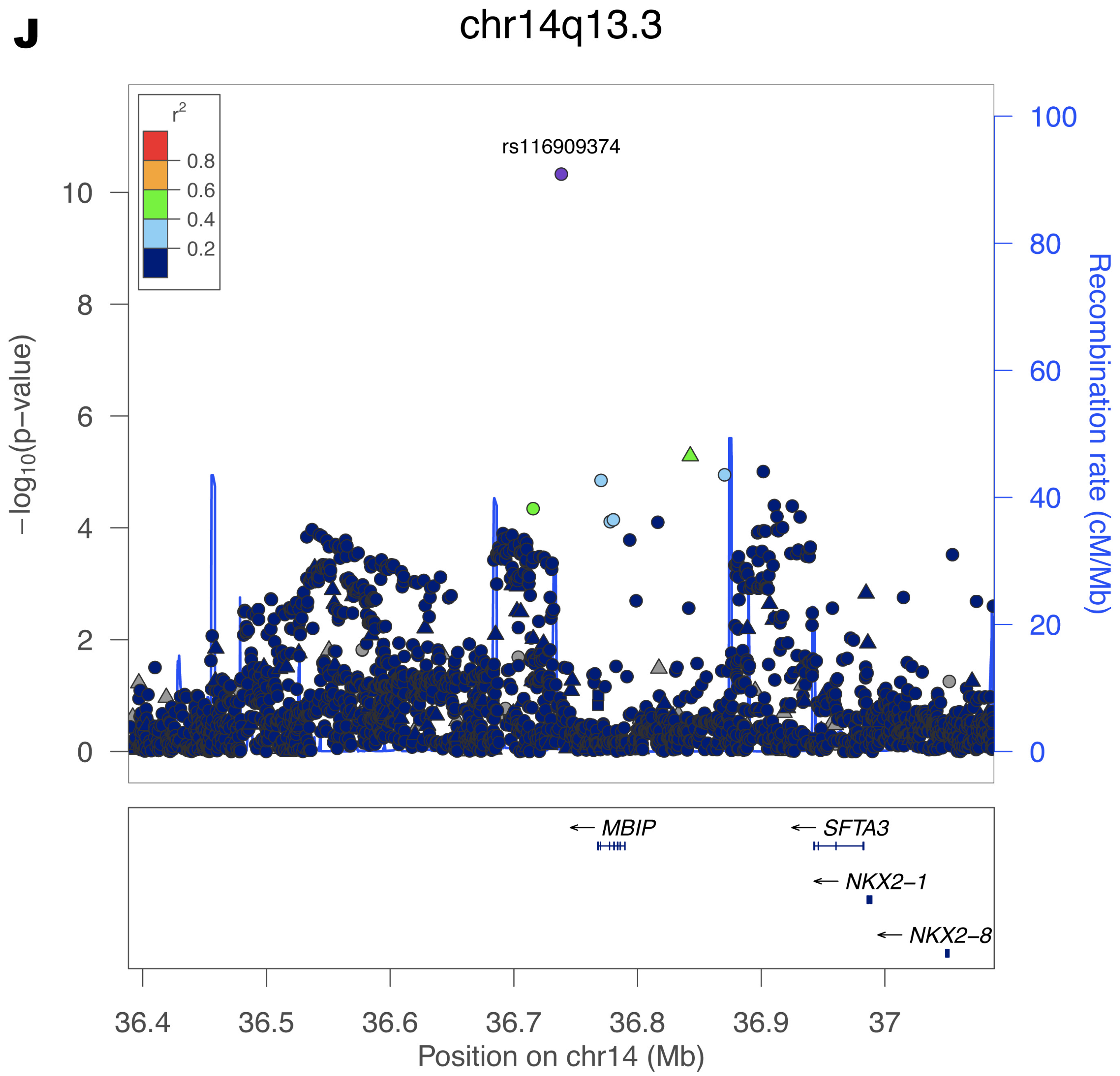
chr14q13.3
r2
0.8
0.6
0.4
0.2
rs116909374
−log10(p−value)
0
2
4
6
8
10
Recombination rate (cM/Mb)
0
20
40
60
80
100
MBIP
SFTA3
NKX2−1
NKX2−8
36.4
36.5
36.6
36.7
36.8
36.9
37
Position on chr14 (Mb)

K

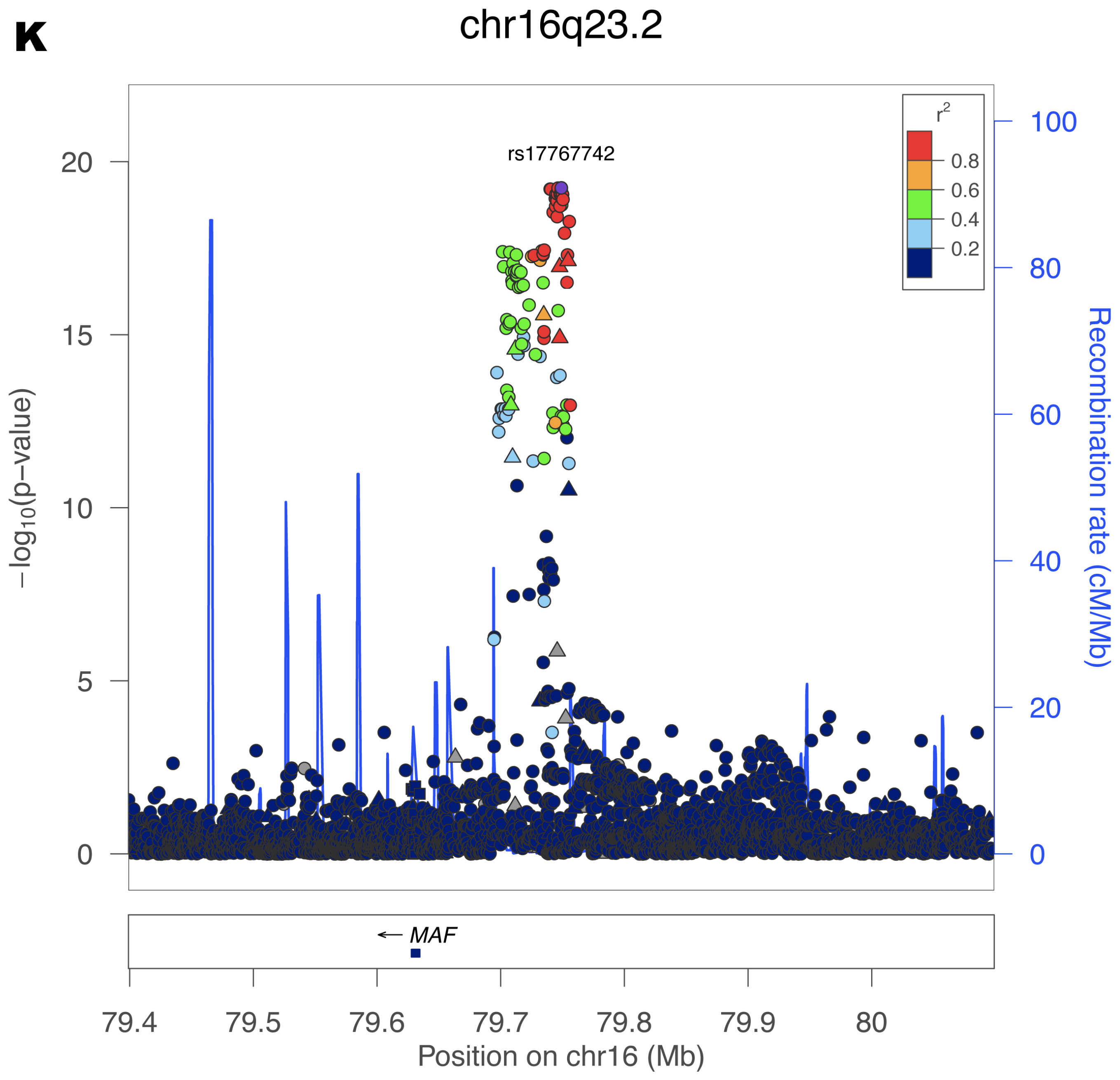

Supplementary Figure 5. Regional association plots showing genome-wide significant loci for serum TSH in the overall meta-analysis. In each panel (A–K), the most significant SNP is indicated (purple circle). The SNPs surrounding the most significant SNP are color-coded to reflect their LD with this SNP as in the inset (taken from pairwise r^2 values from the from the 1000 Genomes reference panel). Symbols reflect genomic functional annotation, as indicated in the legend [1]. Genes and the position of exons, as well as the direction of transcription, are noted in lower boxes. In each panel the scale bar on the Y-axis changes according to the strength of the association.

Supplementary Figure 6. Regional association plots showing genome-wide significant loci for serum FT4 in the overall meta-analysis.

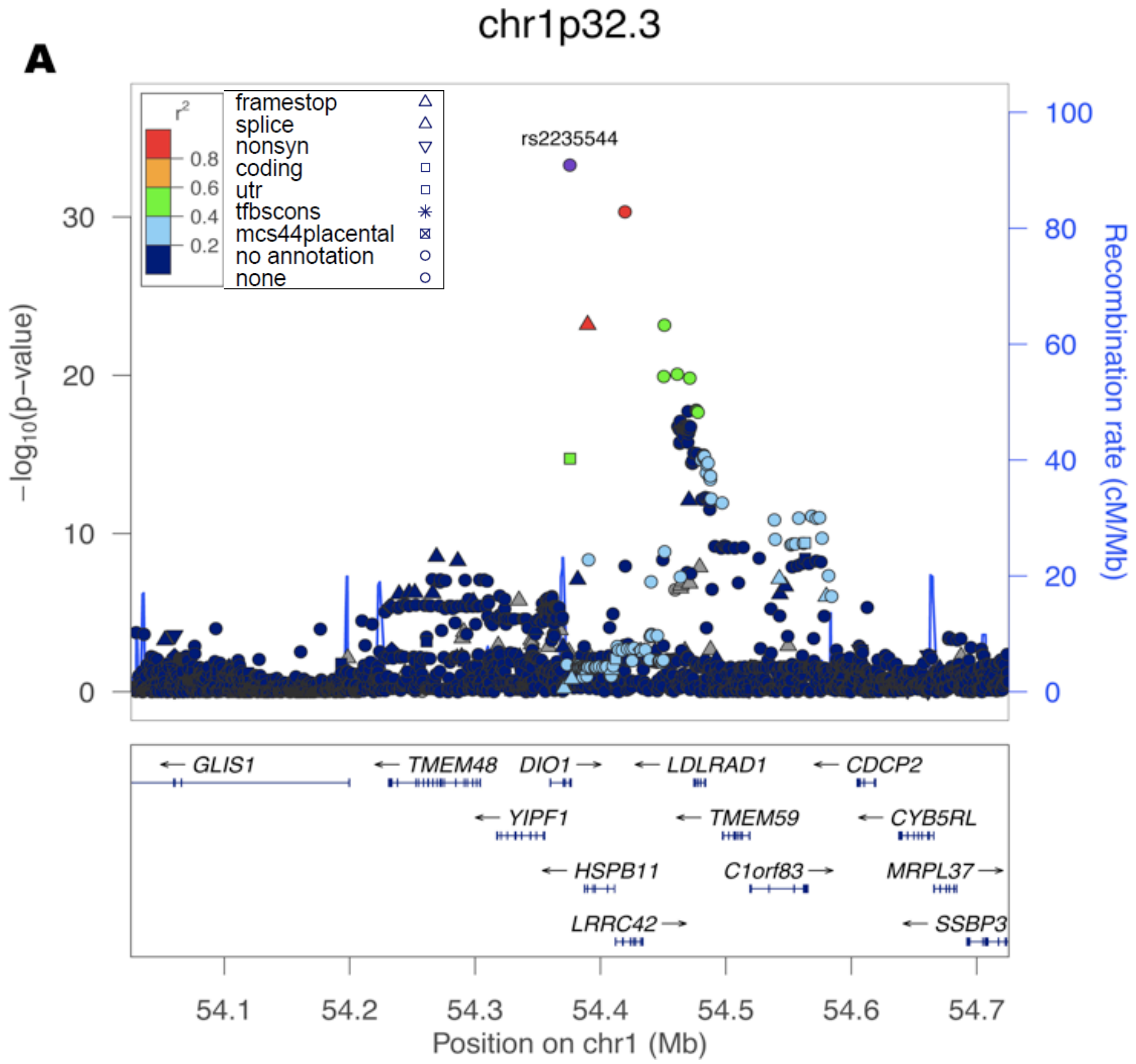

B

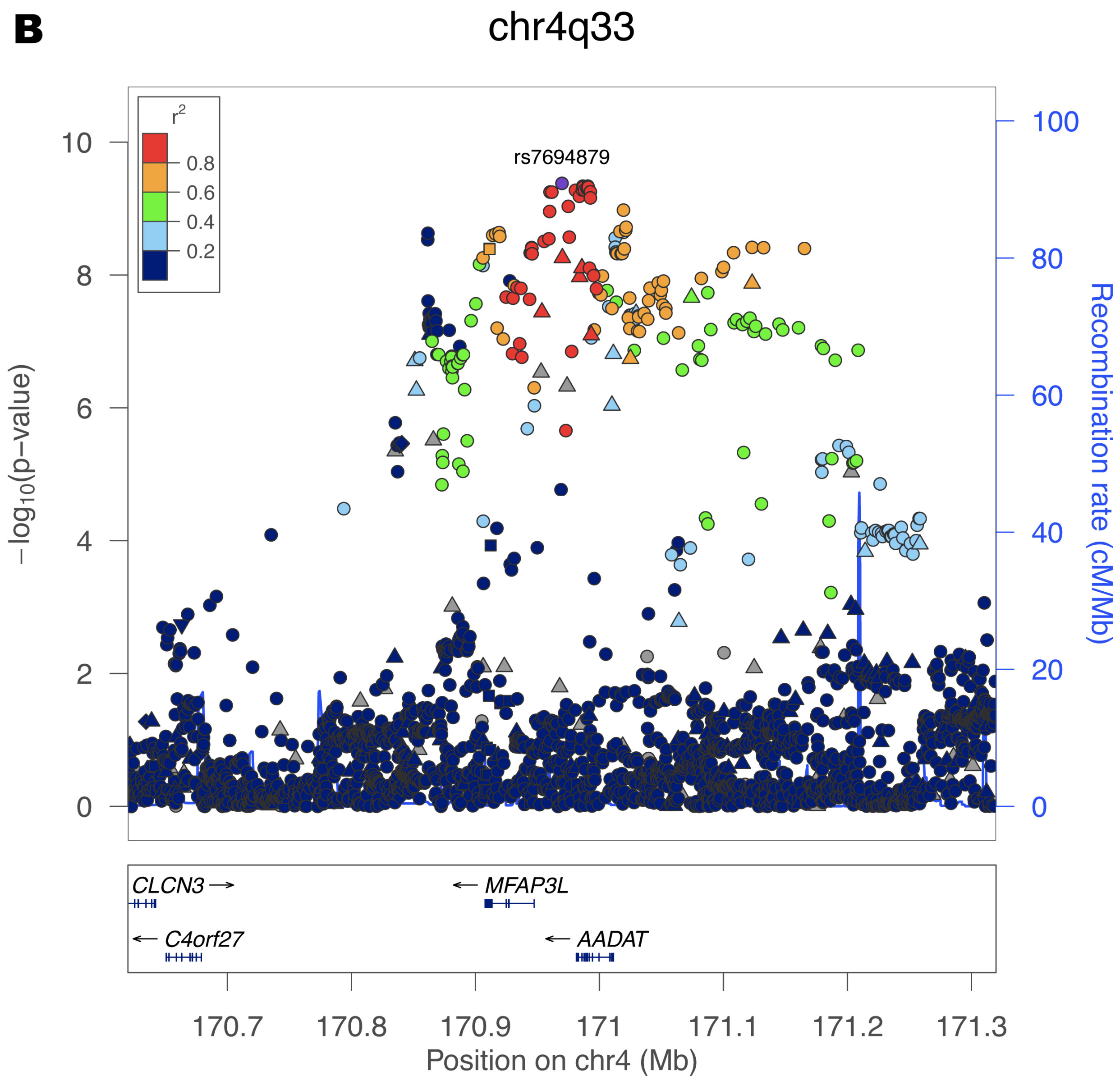
chr4q33
r2
0.8
0.6
0.4
0.2
rs7694879
−log10(p−value)
Recombination rate (cM/Mb)
0
2
4
6
8
10
0
20
40
60
80
100
CLCN3
MFAP3L
C4orf27
AADAT
170.7
170.8
170.9
171
171.1
171.2
171.3
Position on chr4 (Mb)

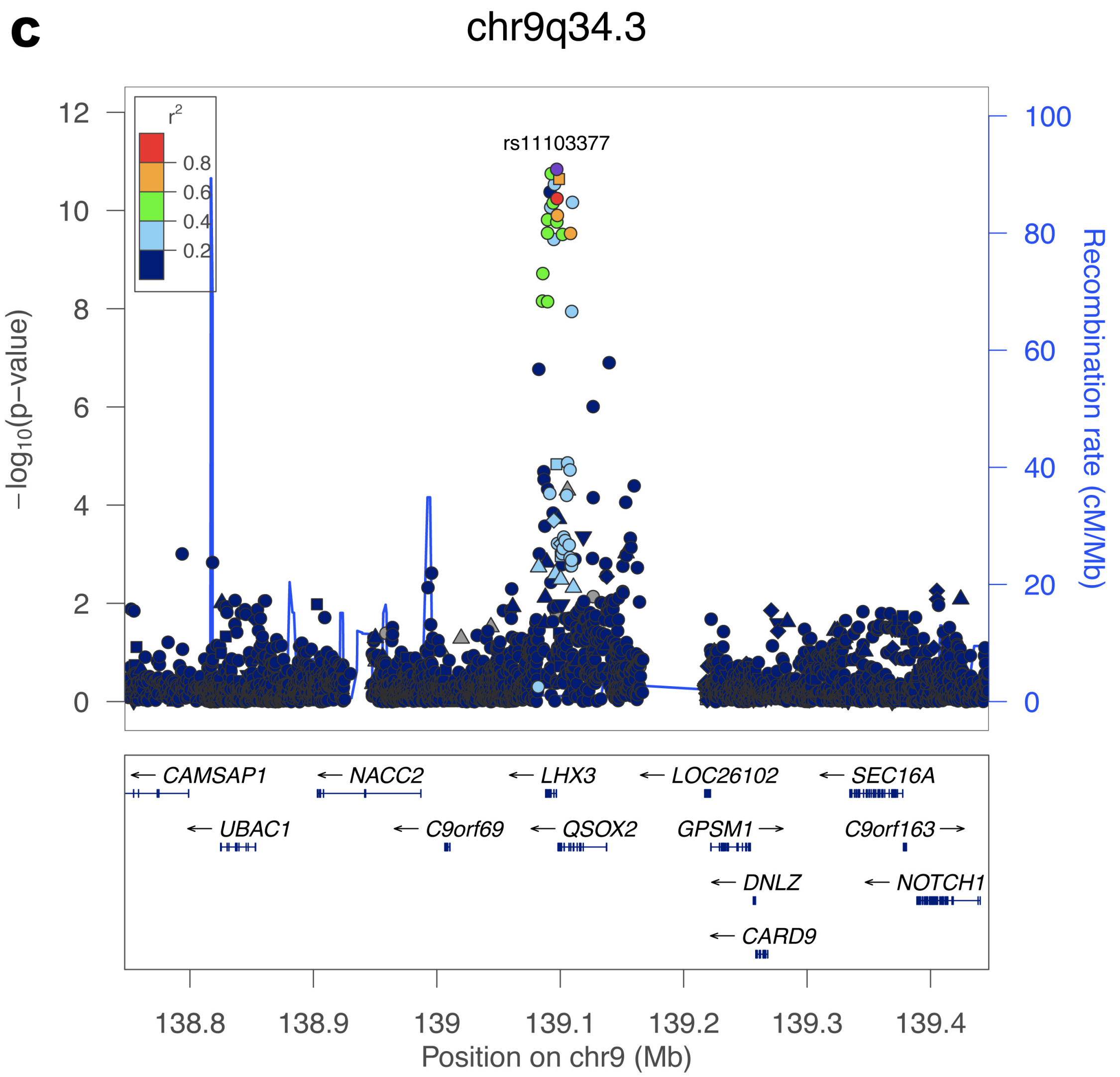

Supplementary Figure 6 Regional association plots showing genome-wide significant loci for serum FT4 in the overall meta-analysis. In each panel (A–C), the most significant SNP is indicated (purple circle). The SNPs surrounding the most significant SNP are color-coded to reflect their LD with this SNP as in the inset (taken from pairwise r^2 values from the from the 1000 Genomes reference panel). Symbols reflect genomic functional annotation, as indicated in the legend [1]. Genes and the position of exons, as well as the direction of transcription, are noted in lower boxes. In each panel the scale bar on the Y-axis changes according to the strength of the association.

Supplementary Tables

Supplementary Table 1. Descriptive statistics for all cohorts included in the study.

Cohort	Subjects (N)	Age, mean (SD)	Age (range)	Male (%)	TSH, mean (SD)	FT4, mean (SD)
TwinsUK WGS	1195	49.8 (12.1)	17-81	0	1.8 (0.8)	1.4 (0.2)
TwinsUK GWAS	1297	47.5 (15.5)	16-82	20.7	1.8 (0.8)	1.4 (0.2)
ALSPAC WGS	1096	7.5 (0.1)	7-9	49.1	2.2 (0.7)	1.6 (0.2)
ALSPAC GWAS	2500	7.6 (0.4)	7-9	53.7	2.1 (0.7)	1.6 (0.2)
SardiNIA	5487	42.8 (17.8)	14-101	47.1	1.7 (0.8)	1.3 (0.2)
ValBorbera	1500	54.1 (17.9)	18-102	46.6	1.5 (0.8)	NA
BHS	3291	52.2 (16.9)	16-97	45.9	1.5 (0.7)	1.7 (0.2)

TSH is reported in mIU/l and FT4 in ng/dl.
SD, standard deviation
BHS, Busselton Health Study

Supplementary Table 2. Cohort Details

	Study	TwinsUK WGS	TwinsUK GWAS	ALSPAC WGS	ALSPAC GWAS	SardiNIA	ValBorbera	BHS
	Ethnicity	European	European	European	European	European	European	European
	Country	UK	UK	UK	UK	Italy	Italy	Australia
	Study design	Twins	Twins	Population-based	Population-based	Family Population-based	Family Population-based	Population-based
TSH MEASUREMENT	Sample type	Serum	Serum	Serum	Serum	Fresh Serum	Fresh Serum	Serum
	Original units	mIU/L	mIU/L	mIU/L	mIU/L	mIU/L	mIU/L	mIU/L
	Assay	Architect, Abbott Diagnostics Division, Modular Analytics E170 Roche Diagnostics	Architect, Abbott Diagnostics Division, Modular Analytics E170 Roche Diagnostics	Cobas® e601 Roche Diagnostics	Cobas® e601 Roche Diagnostics	Siemens, Immulite2000	Beckman DxI 800	Siemens, Immulite2000
	Reference (PMID)	17970774/18681828	17970774/18681828	22956557	22956557	18514160	19847309	20097710
FT4 MEASUREMENT	Sample type	Serum	Serum	Serum	Serum	Fresh Serum	NA	Serum
	Original units	pmol/L	pmol/L	pmol/L	pmol/L	ng/dl	NA	ng/dl
	Assay	Architect, Abbott Diagnostics Division.	Architect, Abbott Diagnostics Division	Cobas® e601 Roche Diagnostics	Cobas® e601 Roche Diagnostics	Siemens, Immulite2000	NA	Siemens, Immulite2000
	Reference (PMID)	17970774	17970774	22956557	22956557	1814160	NA	20097710
PARTICIPANTS	Exclusions	Individuals under hormone replacement therapy, thyroid surgery, TSH<0.4 mIU/L and TSH>4 mIU/L	Individuals under hormone replacement therapy, thyroid surgery, TSH<0.4 mIU/L and TSH>4 mIU/L	Individuals under hormone replacement therapy, thyroid surgery, TSH<0.4 mIU/L and TSH>4 mIU/L	Individuals under hormone replacement therapy, thyroid surgery, TSH<0.4 mIU/L and TSH>4 mIU/L	Individuals under hormone replacement therapy, thyroid surgery, TSH<0.4 mU/L and TSH>4 mU/L	Individuals under hormone replacement therapy, thyroid surgery, TSH<0.4 mIU/L and TSH>4 mIU/L	Individuals taking thyroid medication, TSH<0.4 mIU/L and TSH>4 mIU/L
	Participants with TSH (N, mean, SD)	1195, 1.8, 0.8	1297, 1.8, 0.8	1096, 2.16, 0.74	2,500 2.14, 0.73	5487, 1.70, 0.80	1500, 1.52, 0.75	3291, 1.5, 0.7
	Participants with FT4 (N, mean, SD)	719, 1.4, 0.2	639, 1.4, 0.2	1180, 15.6, 1.72	2638 15.8, 1.68	5487, 1.28, 0.20	NA	3256, 1.7, 0.2
	Age (Mean , SD)	49.8, 12.1	47.5, 15.5	7.5, 0.1	7.6, 0.4	42.80, 17.80	54.1, 17.9	52.2, 16.9
	Male, (N,%)	0 , 0%	268, 20.7%	579, 49.1%	1416, 53.7%	2583, 47.1%	669, 46.6%	1511, 45.9%

GENOTYPING	Genotyping centre	Wellcome Trust Sanger Inst.(UK), BGI (China)	Wellcome Trust Sanger Inst.(UK), CIDR (USA), Centre National de Genotypage (France), Duke University (USA), Helsinki University (Finland)	Wellcome Trust Sanger Inst.(UK), BGI (China)	Wellcome Trust Sanger Institute Cambridge, UK Laboratory Corporation of America, Burlington, NC, USA.	Lanusei, CNR (Sardinia, Italy), Tramariglio, Porto Conte Ricerche (Sardinia, Italy), Baltimore, NIH (USA)	Trieste, Munich	PathWest Laboratory Medicine (Australia), Centre National de Genotypage (France)
	Genotyping/ Sequencing Array	Illumina TruSeq	Illumina Hap300, Hap550, Hap610	Illumina TruSeq	Illumina HumanHap550 quad	Human OmniExpress, Cardio-MetaboChip, ImmunoChip, HumanExome	Illumina SNP array 370K - HumanCNV370-Quadv3 Illumina SNP array 700K -	Illumina 610Q and 660W
	Genotyping calling algorithm	bcftools *bcftools view -m 0.9 -vcgN.*	Illuminus	bcftools *bcftools view -m 0.9 -vcgN.*	Illuminus	GenCall, Zcall (HumanExome)	BeadStudio	Illumina GenomeStudio
SAMPLE GENOTYPING QC	Sample call rate	>95%	>95%	>95%	>95%	>90% (Human OmniExpress), >98% (Cardio-MetaboChip, ImmunoChip, HumanExome)	>95%	>95%
	Individuals for analysis	1195	1297	1096	2,500	6602	1785	4634
SNP QC	MAF (required)	>0%	>1%	> 0%	>1%	>0% (Cardio-MetaboChip, ImmunoChip, HumanExome); >1% (Human OmniExpress)	>=1%	>1%
	HWE (required)	$P>10^{-6}$	$P>10^{-6}$	$P>10^{-6}$	$P>10^{-6}$	$P>10^{-6}$	$P>10^{-4}$	$P>10^{-6}$
	SNPs for imputation	48722573	295702			886938	324326	521307
Imputation	Reference Panel	NA	UK10K/1000 Genomes	NA	UK10K/1000 Genomes	Sardinia	UK10K/1000 Genomes	1000 Genomes
	Build	37	37	37	37	37	37	37
	Software for imputation	BEAGLE 4, rev909	IMPUTE2	BEAGLE 4, rev909	IMPUTE2	Minimac	IMPUTE2	Minimac

	Filters	singletons not overlapping with 1000G	Info score <0.4	singletons not overlapping with 1000G	singletons not overlapping with 1000G	Add this detail	rsqr <0.3, MAF <1%	rsq <0.3
	Variants for analysis (imputed and genotyped)	48722573	25453473	41660141	9558836	9691442	31074568	29777832
Data Analysis	Adjustments	age age2	age age2	age age2 gender	age age2 gender	age age2 gender	age age2 gender	age age2 gender
	Analysis method	Linear regression with additive model for quantitative trait	Univariate linear mixed model accounting for relatedness	Linear regression with additive model for quantitative trait	Univariate linear mixed model	Linear Mixed model adjusted with genomic kinship matrix	polygenic mixed model	Linear regression model for quantitative trait
	Software for analysis	SNPTEST	GEMMA	SNPTEST	SNPTEST	EPACTS	GEMMA	ProbABEL
REFERENCES	Reference study description (PMID)	17254428	17254428	22507743	22507743	16934002	19847309	20097710
	Study link/ website	http://www.twinsuk.ac.uk	http://www.twinsuk.ac.uk	http://www.bristol.ac.uk/alspac/	http://www.bristol.ac.uk/alspac/	http://sardinia.nia.nih.gov/	www.valborbera.org/	www.busseltonhealthstudy.

Supplementary Table 3. Independent SNPs associated with serum TSH levels in the discovery meta-analysis

Gene	SNP	Chromosome	Position	A1/A2	Freq A1	Effect	Std Err	P	N	Het P
**TSH**										
NR3C2	rs117728154	4	149660723	A/G	0.21	-0.21	0.037	8.21×10^{-09}	2285	0.47
FOXE1	rs1877431	9	100534147	A/G	0.40	-0.19	0.030	2.29×10^{-10}	2287	0.07
FAM222A*	rs11067829	12	110116872	G/A	0.18	0.21	0.038	$3.73\mathrm{X}10^{-08}$	2286	0.93

Table shows the association results for SNPs that reached genome-wide level significance in the discovery meta-analysis. For each SNP, the best candidate gene is showed, as well as its genomic position, the effect allele (A1), the other allele (A2), the combined frequency of A1 across studies (Freq A1), the effect size and its standard error (Std Err), the p-value for association (P), the number of samples analyzed (N) and the p-values for heterogeneity of effects across the cohorts (Het P).

*Borderline association P= $<5.0\times10^{-08}$, but $> 1.17\times10^{-08}$

Supplementary Table 4. LD measures between top hits in significant loci from this study and the meta-analysis by Porcu et al[2].

Locus	Gene	Top SNP this study	Top SNP Porcu et al.	LD (r^2)	LD (D')
**TSH**					
1p36	CAPZB	rs12410532	rs10799824	1	1
2q35	IGFBP2/ IGFBP5	rs7568039	rs13015993	0.95	0.99
4q31	NR3C2	rs28435578	rs10032216	0.94	1
5q13	PDE8B	rs2046045	rs6885099	1	1
6p21	VEGFA	rs6923866	rs11755845	0.97	1
6p21	VEGFA	rs2396084	rs9472138	0.82	0.95
6q27	PDE10A	rs3008034	rs753760	1	1
9q34	ABO	rs116552240	rs657152	0.96*	0.99*
14q13	MBIP	rs116909374	rs1537424	0	0.42
16q23	MAF	rs56738967	rs3813582	0.99	1
**FT4**					
1p32	DIO1	rs2235544	rs2235544	NA	NA
4q33	AADAT	rs7694879	rs11726248	0.69	0.87
9q34	LHX3	rs11103377	rs7860634	0.78	0.95

LD values derived from TwinsUK WGS cohort using JLIN software[3]

*LD values derived from 1000 Genomes Project Phase 1 genotype data (EUR population)

Supplementary Table 5. Associations between SNPs displaying heterogeneity in their association with TSH by cohort.

Cohort	A1/A2	MAF	EFFECT	STD ERR	P
***FOXE1* rs7864322**					
TwinsUK Discovery	C/T	0.332	0.120	0.043	0.005
TwinsUK Replication	C/T	0.331	-0.089	0.046	0.053
ALSPAC Discovery	C/T	0.337	0.260	0.044	5.87×10^{-9}
ALSPAC Replication	C/T	0.335	0.240	0.030	1.29×10^{-15}
Sardinia	C/T	0.304	0.055	0.023	0.015
VB	C/T	0.363	0.005	0.039	0.899
Busselton	C/T	0.334	0.058	0.026	0.027
**ABO rs116552240***					
TwinsUK Discovery	C/CA	0.32	-0.02	0.04	0.61
TwinsUK Replication	A/T	0.246	- 0.064	0.056	0.256
ALSPAC Discovery	C/CA	0.34	-0.06	0.044	0.12
ALSPAC Replication	A/T	0.215	-0.062	0.039	0.10
Sardinia	A/T	0.257	-0.200	0.025	5.41×10^{-16}
VB	A/T	0.239	-0.362	0.24	0.14
Busselton	A/T	0.334	-0.072	0.026	0.006
**CAPZB rs12410532**					
TwinsUK Discovery	T/C	0.159	-0.199	0.055	2.82×10^{-4}
TwinsUK Replication	T/C	0.153	-0.194	0.058	0.0008
ALSPAC Discovery	T/C	0.14	0.09	0.06	0.13
ALSPAC Replication	T/C	0.153	0.035	0.039	0.36
Sardinia	T/C	0.181	-0.079	0.027	0.003
VB	T/C	0.156	0.124	0.052	0.018
Busselton	T/C	0.154	-0.111	0.034	0.001
**FAM222A rs11067829**					
TwinsUK Discovery	G/A	0.19	0.21	0.055	4.30×10^{-05}
TwinsUK Replication	G/A	0.17	0.10	0.056	0.07
ALSPAC Discovery	G/A	0.17	0.21	0.056	2.23×10^{-04}
ALSPAC Replication	G/A	0.18	0.01	0.039	0.73
Sardinia	G/A	0.15	0.03	0.025	0.25
VB	G/A	0.20	0.04	0.054	0.47
Busselton	G/A	0.18	0.02	0.038	0.80

*SNP removed from analysis during quality control procedures in both TwinsUK and ALSPAC Cohorts – rs8176646 used as a proxy.

Supplementary Table 6. Details of most likely candidate genes at newly discovered loci for TSH and FT4 levels.

SNP	Gene	Chromosome	Position	Trait	Function
rs310763	*SYN2*	3	12230703	TSH	The synapsin 2 gene encodes neuronal phosphoproteins which link with the cytoplasmic surface of synaptic vesicles and are involved with the modulation of neurotransmitter release; also implicated in several neuropsychiatric diseases [4].
rs2928167	*PDE8B*	5	76477820	TSH	PDE8B encodes a cAMP phosphodiesterase enzyme strongly expressed in the thyroid gland[5], but not the pituitary gland. Another independent locus in this gene has previously been associated with TSH[5]
rs11067829	*FAM222A*	12	110116871	TSH	No relevant data available on this gene/locus.
rs28933981	*TTR*	18	29306736	FT4	TTR is a plasma transport protein for thyroxine and retinol. Mutations in TTR can result in amyloid deposition in peripheral nerves and the heart, resulting in neuropathies and cardiomyopathies [6]. A small portion of TTR mutations are non-amyloidogenic; amongst these are mutations responsible for hyperthyroxinemia, due to a high affinity for thyroxine they may also be protective against familial amyloidotic polyneuropathy amyloid[7,8].
rs113107469	*B4GALT6/ SLC25A52*	18	29306737	FT4	B4GALT6 is in the ceramide metabolic pathway and is also required for the biosynthesis of glycosphingolipids, is heavily expressed in the brain and also the pituitary[9]. Diseases associated with *B4GALT6* include prostate cancer and congenital disorders of glycosylation. The *SLC25A52* gene is similar to the mitochondrial carrier triple repeat 1 gene on chromosome 9 and may be a pseudogene.
SKAT Region	*NRG1*	8		FT4	NRG1 encodes neuoregulin 1, a glycoprotein that interacts with the NEU/ERBB2 receptor tyrosine kinase to increase its phosphorylation on tyrosine residues. NRG1 is a signaling protein that mediates cell-cell interactions and plays a critical role in the growth and development of multiple organ systems. It has previously been associated with TSH[2]. NRG1 gene dysregulation has also been linked to cancer, schizophrenia and bipolar disorder.

Supplementary Table 7. Expression quantitative trait locus analysis (eQTL) for identified variants and proxies in four cell types.

eQTL SNP	Chr: Position	GWAS SNP	LD of eQTL vs. GWAS SNP (r^2)	Candidate Gene	eQTL Gene	Cell type*	Probe	P value
rs1181186	chr1:54292026	rs2235544	0.02‡	DIO1	TMEM48	A	-	N/A
						L	ILMN_1749930	$1.27x10^{-05}$
						S	-	N/A
						W	N/A	N/A
rs11736939	chr4:171031170	rs7694879	0.63†	AADAT	AADAT	A	ILMN_1726986	$7.86x10^{-09}$
						L	-	N/A
						S	ILMN_1726986	$7.85x10^{-04}$
						W	N/A	N/A
rs251429	chr5:76553109	rs2046045	0.35†	PDE8B	PDE8B, WDR41	A	ILMN_1778488 (WDR41)	$1.37x10^{-06}$
						L	ILMN_1778488 (WDR41)	$8.39x10^{-09}$
							ILMN_2301722 (PDE8B)	$9.59x10^{-04}$
						S	ILMN_1778488 (WDR41)	$7.66x10^{-06}$
						W	ILMN_2650148 (WDR41)	$8.69x10^{-27}$
rs10122824	chr9:139109861	rs11103377	0.5†	LHX3	SOHLH1	A	-	-
						L	-	-
						S	ILMN_1656383	$6.17x10^{-05}$
						W	N/A	N/A
rs9462935	chr6:43828573	rs2396084	0.54†	VEGFA	MRPL14	A	ILMN_2072603	$4.56x10^{-04}$
						L	-	-
						S	-	-
						W	N/A	N/A
rs310763	chr3:12230704	rs310763	N/A	SYN2	SYN2, TIMP4	A	ILMN_1781060 (SYN2)	$4.27x10^{-17}$
							ILMN_1663399 (TIMP4)	$8.55x10^{-09}$
						L	-	-
						S	ILMN_1781060 (SYN2)	$2.57x10^{-12}$
						W	ILMN_3420044 (SYN2)	$3.18x10^{-36}$
rs3758249	chr9:100614140	rs13295254	0.25†	FOXE1	C9orf156	A	ILMN_1700028	$1.93x10^{-09}$
						L	-	-
						S	ILMN_1700028	$7.15x10^{-11}$
						W	ILMN_3440446	$9.10x10^{-54}$

†LD derived from 1000 Genomes Project ‡LD derived from TwinsUK WGS cohort * Cell types: A adipose cell, L lymphoblastoid cell line, S skin cell W whole blood. N/A Not available

Supplementary Table 8. DNA methylation quantitative trait locus (meQTL) analysis results at the thyroid-associated variants from Table 1.

SNP	CpG-site	CpG chr:position	CpG distance to SNP	Candidate gene	CpG distance to candidate gene TSS	meQTL results				
						WGS P	GWAS P	Meta-analysis		ALSPAC
								B (SE)	P	P
**TSH**										
rs2928167	cg16418800	5:76476456	1364	PDE8B	30250	1.88x10^{-5}	7.85x10^{-03}	-0.61 (0.12)	4.38x10^{-07}	3.03x10^{-28}
rs6923866	cg18120259	6:43894639	6545	VEGFA	149179	1.12x10^{-3}	1.74x10^{-03}	-0.41 (0.09)	4.06x10^{-06}	-
rs2396084	cg11772020	6:43806470	1645	VEGFA	61010	2.00x10-^{4}	0.03	-0.36 (0.08)	1.45x10^{-05}	-
rs116552240	cg11879188	9:136149908	810	ABO	722	3.06x10^{-6}	3.77x10^{-06}	0.72 (0.10)	4.35x10^{-12}	6.65x10^{-34}
	cg21160290	9:136149941	843	ABO	689	2.18x10^{-10}	2.22x10^{-09}	0.91 (0.10)	6.94x10^{-22}	1.01x10^{-101}
	cg22535403	9:136150032	934	ABO	598	3.03x10^{-11}	1.38x10^{-09}	0.92 (0.09)	2.02x10^{-23}	8.91x10^{-100}
	cg24267699	9:136151359	2261	ABO	729	9.05x10^{-05}	3.17x10^{-05}	0.69 (0.12)	2.53x10^{-09}	5.40x10^{-92}
**FT4**										
rs7694879	cg24693803	4:171013537	43738	AADAT	2165	1.03x10^{-04}	1.13x10^{-04}	0.81 (0.14)	1.80x10^{-08}	4.18x10^{-34}

Supplementary Table 9. Results of top ranked p-values for the sequence kernel based association tests (SKAT) meta-analysis for FT4.

Trait	Gene	Chromosome	Bin	P-value	Pmin	Cmaf (%)	NSNPs
**Meta-analysis**							
FT4							
	NRG1	8	117	3.01×10^{-04}	3.04×10^{-04}	4.45	24
	NRG1	**8**	**118**	$\mathbf{2.53\times10^{-06}}$	$\mathbf{1.39\times10^{-04}}$	**5.29**	**19**
	TTR	18	24	4.94×10^{-05}	1.76×10^{-06}	2.85	32
	B4GALT6	14	14	1.20×10^{-04}	1.00×10^{-06}	2.19	30
	NETO1	18	1	1.03×10^{-03}	8.03×10^{-04}	4.76	34
	NETO1	18	6	8.83×10^{-04}	7.76×10^{-04}	6.09	21

$P < 1.55\times10^{-5}$ is regarded as the multiple testing corrected (Bonferroni) significance threshold.
There were no observed SKAT associations with TSH
Cmaf - Cumulative minor allele frequency
NSNPs – Number of SNPs

Supplementary Table 10. Percentage of variance in TSH and FT4 explained by genetic variants.

		**All SNPs maf>0.01**		**Known SNPs Previous Studies**		**Known SNPs + Novel Top Hits**	
	N_{TSH}, N_{FT4}	TSH (SD)	FT4 (SD)	TSH (SD)	FT4 (SD)	TSH (SD)	FT4 (SD)
ALSPAC	1092, 1088	21.8 (3.4)<br>p=0.2657	16.3 (17.6)<br>p=0.178	9.3 (2.8)<br>$p=1.67x10^{-16}$	4.6 (1.9)<br>$p=1.58x10^{-5}$	10.4 (2.9)<br>$p= x10^{-06}$	4.7 (1.9)<br>$p=1.69x10^{-05}$
TWINS UK	1195, 719	86.6 (29.8)<br>p=0.0013	15.4 (50.5)<br>p=0.381	6.9 (2.3)<br>$p=7.37x10^{-11}$	2.2 (1.2)<br>p=0.0458	8.2 (2.5)<br>$p=3.84x10^{-13}$	3.4 (1.9)<br>p=0.00839
SardiNIA	5487, 5487<br>(1790,1790)	21.5 (3.3)<br>$p = 1x10^{-16}$	13.1 (0.038)<br>$p = 1x10^{-16}$	9.4 (2.5)<br>$p=1x10^{-16}$	1.6(0.86)<br>p=0.0053	9.9 (2.5)<br>$p=1x10^{-16}$	2.1 (0.98)<br>p=0.00085
BHS	3291, 3256	0.277(0.048)<br>$p=5.88x10^{-10}$	0.33 (0.049)<br>$p=4.50x10^{-13}$	0.057 (0.016)<br>$p < 1x10^{-16}$	0.016 (0.007)<br>$p = 3.64x10^{-06}$	0.063 (0.017)<br>$p = 1x10^{-16}$	0.015<br>(0.0063)<br>$p = 8.3x10^{-06}$
Meta<br>ALSPAC<br>+TWINSUK	2287, 1807	0.56 (0.32) R<br>0.58 (0.22) F<br>p = 0.082/<br>0.009	0.16 (0.17) R<br>0.16 (0.17) F<br>p=0.330	0.079 (0.018)<br>p < 0.0001	0.032 (0.012)<br>p = 0.009	0.091 (0.019)<br>p < 0.0001	0.041 (0.013)<br>p = 0.0023
Meta All		0.26 (0.052) R<br>0.24 (0.027) F<br>p < 0.0001	0.22 (0.078) R<br>0.20 (0.030) F<br>p=0.0057/<br>< 0.0001	0.072 (0.011)<br>p < 0.0001	0.018<br>(0.0047)<br>p = 0.0001	0.080 (0.011)<br>p < 0.0001	0.021<br>(0.0054)<br>0.020<br>(0.0049)<br>p=0.0001/<br><0.0001

MAF, Minor allele frequency
N, Number of samples analyzed
VE, Variance explained
95% CI, 95 % confidence interval
P, p-value for association
Random effects estimate, F = fixed effects estimate. Where R/F is not indicated for meta-analysis, results were identical for random and fixed effects analyses.
*Only HWE p-value > 0.05 SNPs used
**ALSPAC and TUK are the WGS cohorts.

Supplementary Table 11. Rank of genes associated with thyroid function in the levothyroxine responsiveness cell-based studies.

Gene	SNP	probe_id	4069_PC3	4150_MCF7	1312_HL60
CAPZB	rs12410532	215097_at	198**	20277	11072
SYN2	rs310763	210247_at	5756	4211	64***
NRG1	rs7825175	208230_s_at	610*	684*	17552

Numbers are the rank in responsiveness among a total number of 22,283 probes, ***P<0.005, **P<0.01, *P<0.05

Supplementary Methods

Cohort descriptions

Twins UK: The Twins UK cohort consists of 12,000 twins of northern European/UK ancestry, aged 16–82 yr, from St Thomas' UK Adult Twin Registry (TwinsUK), a volunteer sample recruited in the United Kingdom without selection for particular traits (www.twinsuk.ac.uk/). It has previously been shown to be representative of singleton populations and the UK population in general[10].

ALSPAC: ALSPAC is a geographically based UK cohort that recruited pregnant women residing in Avon (Southwest England) with an expected date of delivery between April 1, 1991, and December 31, 1992. A total of 15,247 pregnancies were enrolled, with 14,775 children born (see www.alspac.bris.ac.uk.)[11]. Please note that the study website contains details of all the data that is available through a fully searchable data dictionary" http://www.bris.ac.uk/alspac/researchers/data-access/data-dictionary/. The ALSPAC cohort was the only child cohort used in this study however both the hypothalamic-thyroid axis and the impact of thyroid status on metabolism is considered to be generally comparable between children and adults[12,13].

SardiNIA: The SardiNIA study consists of the former SardiNIA study (6,148 volunteers) previously described[14], and additional 588 individuals enrolled during the follow up stage. They are in part relatives of individuals already enrolled in the study, or new families living in the four villages involved in the project that agreed to participate only recently[15].

ValBorbera (INGI): The Val Borbera (INGI) population is a collection of 1,785 genotyped samples collected in the Val Borbera Valley, a geographically isolated valley located within the Appennine Mountains in NorthWest Italy[16]. The valley is inhabited by about 3000 descendants from the original population, living in 7 villages along the valley and in the mountains. The valley was inhabited by about 10,000 people in the 19th century when endogamy was >80%. Participants were healthy people between 18 and 102 years of age that had at least one grandfather living in the valley. The study was approved by the San Raffaele Hospital Ethical committee and all participants provided a written informed consent.

Busselton: The Busselton Health Study (http://bsn.uwa.edu.au) includes a series of cross-sectional health surveys carried out since 1966 of residents of Busselton, a rural town with a predominantly Caucasian population, located in the southwest of Western Australia[17]. In 1994-5, there was a follow-up study of people who had participated in previous studies. Participants completed a health questionnaire, underwent physical examination, and gave a venous blood sample in the morning after an overnight fast.

Cohort Exclusions

Each participating study excluded individuals with known thyroid disease, those receiving thyroid medication, those who had previously undergone thyroid surgery those who had TSH <0.4 mIU/l or a TSH >4.0 mIU/l or FT4 values outside of the relevant reference range or those who had failed genotyping QC.

Cohort Acknowledgements

UK10K: Whole genome sequencing was performed by UK10K Consortium (http://www.uk10k.org/studies/cohorts.html). UK10K received funding support from the Wellcome Trust. N.S.'s research is supported by the Wellcome Trust (grant codes WT098051 and WT091310), the EU FP7 (EPIGENESYS grant code 257082 and BLUEPRINT grant code HEALTH-F5-2011-282510).

TwinsUK: The TwinsUK study is grateful to the volunteer twins who made available their time. We thank the staff from the genotyping facilities at the Wellcome Trust Sanger Institute and the Center for Inherited Disease Research as part of a National Eye Institute/National Institutes of Health project grant. We gratefully acknowledge the contribution of Abbott Diagnostics, North Ryde, Australia and Roche Diagnostics, Australia which provided support for the biochemical analysis. This study received funding from the Wellcome Trust; the European Community's Seventh Framework Program grant agreement (FP7/2007-2013); ENGAGE project grant agreement (HEALTH-F4-2007-201413); the Department of Health via the National Institute for Health Research (NIHR) Comprehensive Biomedical Research Centre award to Guy's & St Thomas' NHS Foundation Trust in partnership with King's College London; the Canadian Institutes of Health Research, Canadian Foundation for Innovation, Fonds de la Recherche en Santé Québec, Ministère du Développement Économique, de l'Innovation et de l'Exportation Québec and the Lady Davis Institute of the Jewish General Hospital (JBR); the Australian National Health and Medical Research Council (Project Grants 1010494, 1031422, 1048216) and the Sir Charles Gairdner Hospital RAC (JW, SGW); the iVEC supercomputing facilities (EPIC, Murdoch University, Western Australia). The study also receives support from the National Institute for Health Research (NIHR)- funded BioResource, Clinical Research Facility and Biomedical Research Centre based at Guy's and St Thomas' NHS Foundation Trust in partnership with King's College London. SNP Genotyping was performed by The Wellcome Trust Sanger Institute and National Eye Institute via NIH/CIDR. T.D.S. is an NIHR senior investigator. We also thank Mr Chris Bording, iVEC Supercomputing Specialist and Developer, for assistance with bioinformatics aspects of the study

ALSPAC: ALSPAC are extremely grateful to all the families who took part in this study, the midwives for their help in recruiting them, and the whole ALSPAC team, which includes interviewers, computer and laboratory technicians, clerical workers, research scientists, volunteers, managers, receptionists and

nurses. The UK Medical Research Council, The Wellcome Trust (grant number 092731) and the University of Bristol provide core support for ALSPAC. Thyroid function was performed from grants obtained from the BUPA research foundation, the British Thyroid Foundation and the Above and Beyond charitable trust. P.T is funded through the Welsh Clinical Academic Training scheme. Ethical approval for the study was obtained from the ALSPAC Ethics and Law Committee and the Local Research Ethics Committees.

SardiNIA: The SardiNIA study thanks the many individuals who generously participated in this study, Monsignore Piseddu, Bishop of Ogliastra, the mayors and citizens of the Sardinian towns (Lanusei, Ilbono, Arzana, and Elini), and the head of the Public Health Unit ASL4 for their volunteerism and cooperation; the team also thanks the physicians Marco Orrù, Maria Grazia Pilia, Liana Ferreli, Francesco Loi, nurses Paola Loi, Monica Lai and Anna Cau who carried out participant physical exams, the recruitment personnel Susanna Murino, Michele Marongiu for informatic support. We also thank Fabio Busonero, Antonella Mulas, Mariano Dei, Sandra Lai, Andrea Maschio for genotyping and Monia Lobina for DNA and serum extraction. This work was supported by the Intramural Research Program of the National Institute on Aging (NIA), National Institutes of Health (NIH), with contracts NO1-AG-1–2109 and HHSN271201100005C; by Italian grants FISM 2011/R/13 "Approccio razionale per la ricerca di composti per la cura della sclerosi multipla basato sull'analisi dei target biologici individuati dagli studi di associazione sull'intero genoma in Sardegna", FaReBio2011 "Farmaci e Reti Biotecnologiche di Qualità", Funds MIUR/CNR for rare diseases and molecular screening, PNR-CNR Aging Program 2012-2014. The efforts of EP were supported in part by contract 263-MA-410953 from the NIA to the University of Michigan and by research grant HG002651 and HL084729 from the NIH.

ValBorbera: The ValBorbera study thanks the inhabitants of the Val Borbera for participating in the study, the local administrations and the ASL-Novi Ligure and the San Raffaele Hospital for support. We thank Fiammetta Viganò for technical help and Prof. Paolo Beck-Peccoz, Luca Persani and Laura Fugazzola (University of Milano, Milano, Italy) for thyroid clinical analysis. The research was supported by funds from Compagnia di San Paolo, Torino, Italy; Fondazione Cariplo, Italy; Telethon Italy; Ministry of Health, Ricerca Finalizzata 2007 and Public Health Genomics (CCM) Project 2010; PRIN 2009.

Busselton: We thank the Busselton Population Medical Research Foundation for permission to access the survey data and stored sera, and Kashif Mukhtar and Professor Matthew Knuiman for help in accessing samples, data extraction, and advice. We also thank Siemens Healthcare Diagnostics for donating assay kits, the Australian National Health and Medical Research Council (Project Grant 1031422) and the Sir Charles Gairdner Hospital RAC (JW, SGW)

UK10K genetic analysis

Sequence data production

For the UK10K project, low coverage WGS was performed at both the Wellcome Trust Sanger Institute (WTSI) and the Beijing Genomics Institute (BGI). DNA (1-3µg) was sheared to 100–1000 bp using a Covaris E210 or LE220 (Covaris, Woburn, MA, USA). Sheared DNA was size subjected to Illumina paired-end DNA library preparation. Following size selection (300-500 bp), DNA libraries were sequenced using the Illumina HiSeq platform as paired-end 100 base reads according to manufacturer's protocol.

Alignment and BAM processing

Data generated at the WTSI and BGI were aligned to the human reference separately by the respective centres. The BAM files[18] produced from these alignments were submitted to the European Genome-phenome Archive (EGA). The Vertebrate Resequencing Group at the WTSI then performed further processing.

Alignment

Sequencing reads that failed QC were removed using the Illumina GA Pipeline, and the rest were aligned to the GRCh37 human reference, specifically the reference used in Phase 1 of the 1000 Genomes Project (1000GP)[19] (ftp://ftp.1000genomes.ebi.ac.uk/vol1/ftp/technical/reference/human_g1k_v37.fasta.gz). Reads were aligned using BWA (v0.5.9-r16)[20]. This involved the following steps:

1. Index the reference fasta file:

    bwa index -a bwtsw <reference_fasta>

2. For each fastq file:

    bwa aln -q 15 -f <sai_file> <reference_fasta> <fastq_file>

3. Create SAM files [sam] using bwa sampe for paired-end reads:

    bwa sampe -f <sam_file> <reference_fasta> <sai_files> <fastq_files>

4. Create sorted BAM from SAM. For alignments created at the WTSI this was done using Picard (v1.36) (http://picard.sourceforge.net/) SamFormatConverter and samtools (v0.1.11) sort. For alignments created at the BGI, this was done using samtools (v0.1.8) view and samtools sort.
5. PCR duplicates reads in the WTSI alignments were marked as duplicate using the Picard MarkDuplicates, while in the BGI alignments they were removed using samtools rmdup.

BAM improvement and sample file production

Further processing to improve SNV and INDEL calling, including realignment around known INDELs, base quality score recalibration, addition of BAQ tags, merging and duplicate marking follows that used for

Illumina low-coverage data in 1000GP. Software versions used for UK10K for the steps described in that section were GATK version 1.1-5-g6f43284, Picard version 1.64 and samtools version 0.1.16.

Variant calling

SNV and INDEL calls were made using samtools/bcftools (version 0.1.18-r579)[21] by pooling the alignments from 3,910 individual low read-depth BAM files. All-samples and all-sites genotype likelihood files (bcf) were created with the samtools mpileup command

samtools mpileup -EDVSp -C50 -m3 -F0.2 -d 8000 -P ILLUMINA -g –f <reference_fasta>

with the flags

C=Coefficient for downgrading mapping quality for reads containing excessive mismatches

d=At a position, read maximally d reads per input BAM.

Variants were then called using the following bcftools command to produce a VCF file [22]

bcftools view -m 0.9 -vcgN.

For calling on chromosome X and Y, the following settings were applied. The pseudo-autosomal region (PAR) was masked on chromosome Y in the reference fasta file. Male samples were called as diploid in the PAR on chromosome X, and haploid otherwise. Diploid/haploid calls were made using the -s option in bcftools view. The PAR regions were: X-PAR1 (60,001-2,699,520); X-PAR2 (154,931,044-155,260,560); Y-PAR1 (10,001-2,649,520); Y-PAR2 (59,034,050-59,363,566). The pipeline (run-mpileup) used to create the calls is available from https://github.com/VertebrateResequencing/vr-codebase/tree/develop.

Filtering

INDEL pre-filtering

The observation of spikes in the insertion/deletion ratio in sequencing cycles of a subset of the sequencing runs were linked to the appearance of bubbles in the flow cell during sequencing. To counteract this, the following post-calling filtering was applied. The bamcheck utility from the samtools package was used to create a distribution of INDELs per sequencing cycle. Lanes with INDELs predominantly clustered at certain read cycles were marked as problematic, specifically where the highest peak was 5x bigger than the median of the distribution. The list of problematic lanes included 159 samples. In the next step we checked mapped positions of the affected reads to see if they overlapped with called INDELs, which they did for 1,694,630 called sites. The genotypes and genotype likelihoods of affected samples were then set to the reference genotype unless there was a support for the INDEL also in a different, unaffected lane from the

same sample. In total, 140,163 genotypes were set back to reference and 135,647 sites were excluded by this procedure. Note that this step was carried out on raw, unfiltered calls prior to VQSR filtering.

Site filtering

Variant Quality Score Recalibration (VQSR) [23] was used to filter sites. For SNVs, the GATK (version 1.3-21) UnifiedGenotyper was used to recall the sites/alleles discovered by samtools in order to generate annotations to be used for recalibration. Recalibration for the INDELs used annotations derived from the built-in samtools annotations. The GATK VariantRecalibrator was then used to model the variants, followed by GATK ApplyRecalibration, which assigns VQSLOD (variant quality score log odds ratio) values to the variants. SNVs and INDELs were modeled separately, with parameters given below:

	SNVs	INDELs
Annotations	QD, DP, FS, MQ, HaplotypeScore, MQRankSum, ReadPosRankSum, InbreedingCoeff	MSD, MDV, MSQ, ICF, DP, SB, VDB
Training set	HapMap 3.3: hapmap_3.3.b37.sites.vcf, Omni 2.5M chip: 1000G_omni2.5.b37.sites.vcf	Mills-Devine [24], 1000 Genomes Phase I
Truth set	HapMap 3.3: hapmap_3.3.b37.sites.vcf	Mills-Devine
Known set	dbSNP build 132: dbsnp_132.b37.vcf	Mills-Devine

The truth set included sites defined as truly showing variation from the reference (GRCh37). VQSLOD scores were calibrated by how many of the truth sites were retained when sites with a VQSLOD score below a given threshold were filtered out. For SNV sites a truth sensitivity of 99.5%, which corresponded to a minimum VQSLOD score of -0.6804, was selected (i.e. for this threshold 99.5% of truth sites were retained). For INDEL sites a truth sensitivity of 97%, which corresponded to a minimum VQSLOD score of 0.5939, was chosen. Post VQSLOD filtering, we also introduced the filter $p<10^{-6}$ to remove sites that failed Hardy-Weinberg equilibrium (HWE, 302,388 sites removed). Finally, logistic regression models were fitted for the whole genotype set where each sample was characterised according to its cohort and sequencing centre (BGI vs WTSI). We removed sites with evidence for differential frequency (logistic regression $p>10^{-2}$) between samples sequenced at BGI and WTSI (277,563 sites removed) After removal of batch effects, we re-computed the pairwise IBS metrics using an LD-pruned genotype set and performed a multidimensional scaling analysis (MDS) on 10 dimensions (PLINK,v1.07), which confirmed the removal of the original structure. We note that the batch-effect correction applied in this case is over-conservative. However, as shown in the QQ plots inflation of summary statistics is well controlled in all tests applied.

The final data set includes the VQSLOD score and other annotations from GATK (BaseQRankSum, Dels, FS, HRun, HaplotypeScore, InbreedingCoeff, MQ0, MQRankSum, QD, ReadPosRankSum, culprit), but it excludes annotations that already existed in or did not apply to the samtools VCFs (DP and MQ, AC, AN). Each VCF further contained the filters LowQual (a low quality variant according to GATK) and MinVQSLOD (variant's VQSLOD score is less than the cutoff). All sites that did not fail these filters were marked as PASS and brought forward to the genotype refinement stage.

Post-genotyping sample QC

Of the 4,030 samples (1,990 TwinsUK and 2,040 ALSPAC) that were submitted for sequencing, 3,910 samples (1,934 TwinsUK and 1,976 ALSPAC) were sequenced and went through the variant calling procedure. Low quality samples were identified before the genotype refinement by comparing the samples to their GWAS genotypes [25] using about 20,000 sites on chromosome 20. Comparing the raw genotype calls to these existing GWAS genotypes data, we removed a total of 112 samples (64 TwinsUK and 48 ALSPAC) because of one or more of the following causes: (i) high overall discordance to GWAS genotype data (>3%) (55 TwinsUK and 36 ALSPAC), (ii) heterozygosity rate > 3SD from population mean (1 TwinsUK and 1 ALSPAC), suggesting possible contamination (iii) no GWAS genotype data available for that sample (7 TwinsUK and 0 ALSPAC) and (iv) sample below 4x mean read-depth (1 TwinsUK and 11 ALSPAC). Overall, 3,798 samples (1,870 TwinsUK and 1,928 ALSPAC) were brought forward to the genotype refinement step.

Genotype refinement

The missing and low confidence genotypes in the filtered VCFs were refined through an imputation procedure with BEAGLE 4, rev909 [26]. The program was run with default parameters. VCFs were split into chunks each containing a maximum of 3,000 sites plus 1,000 sites in buffer regions, that is 500 on either side. Multiallelic sites were included in the imputation. It took 882 CPU weeks to complete. After imputation, chunks were recombined using the vcf-phased-join script from the vcftools [22] package.

Post-refinement sample QC

Additional sample-level QC steps were carried out on refined genotypes, leading to the exclusion of additional 17 samples (16 TwinsUK and 1 ALSPAC) due to one or more of the following causes: (i) post-refinement non-reference discordance (NRD) with GWAS data > 5% (12 TwinsUK and 1 ALSPAC), (ii) multiple relations to other samples, i.e. more than 25 relations with IBS>0.125 were deemed indicative of contamination (13 TwinsUK and 1 ALSPAC), (iii) failed sex check (3 TwinsUK and 0 ALSPAC). To identify these samples we pruned the WGS data to a set of independent SNVs and calculated genome-wide

average identity by state between each pair of samples across the two cohorts. The resulting set of contaminated samples corresponded almost completely to the set of samples with NRD>5%. This left a final set of 3,781 samples (1,854 TwinsUK and 1,927 ALSPAC).

Re-phasing

SHAPEIT2 [27] was then used to rephase the genotype data. The VCF files were converted to binary ped format. Multiallelic and MAF<0.02% (singleton and monomorphic) sites were removed. Files were then split into 3Mbp chunks with +/-250kbp flanking regions. SHAPEIT (v2.r727) was used to rephase the haplotypes with the following command line option in phase mode:

```
--thread 4 --window 0.5 --states 200 --effective-size 11418 -B chr20.$chunk --input-map genetic_map_chr20_combined_b37.txt --output-log $log --output-max chr20.$chunk.hap.gz chr20.$chunk.sample
```

vcf-gensample [vcftools] was used to combine the original VCF with new phase information. Sites not rephased with SHAPEIT had any existing phase information removed. vcf-phased-join was used to stitch the chunked VCFs back together with phase determined by matching overlapping heterozygous sites.

These are the final VCF files released for the project and submitted to the EGA. An imputation reference panel in the IMPUTE2 format created from these VCF files are also made available.

Removal of sequencing centre batch effects

To investigate the presence of batch effects between sequencing centres in the cohorts dataset (WTSI and BGI), we computed pairwise IBS metrics for a joint dataset of 3,621 individuals using an LD-pruned genotype set of 2,203,581 markers and performed a multidimensional scaling analysis (MDS) on 10 dimensions (PLINK,v1.07, options: --indep-pairwise, window size: 5000 SNVs, step: 1000 SNVs, r^2: 0.2; --mds-plot 10). Each sample was labelled by cohort and sequencing centre. Case/control status was assigned to each individual based on their sequencing centre ("BGI" vs "SANGER") and logistic regression models were applied to test for differences in allele frequency between the two centres, with cohort of origin ("ALSPAC" and "TwinsUK") treated as covariate. Here we used the whole genotype set (MAF≥1%, 46,857,518 SNPs). A total of 335,982 SNPs SNVs displayed significant association with sequencing centre (p-value ≤ 0.01) and were thus removed from analysis, removing batch effects between the two sequencing centres.

Removal of related and non-European ancestry samples for association analyses

The final release set that passed all QC contains non-European and related samples, both of which we sought to exclude to simplify the association testing. To identify participants of non-European ancestry we merged a pruned dataset to the 11 HapMap3 populations [28] and performed a principal components analysis (PCA) using EIGENSTRAT [29]. A total of 44 participants (12 TwinsUK and 32 ALSPAC) did not cluster to the European (CEU) cluster of samples and were removed from association analyses. We further sought to flag related individuals for exclusion in association tests. Overall, 69 samples (36 TwinsUK and 33 ALSPAC) were flagged because of relatedness greater than third degree relatedness (IBS>0.125). Finally 63 co-twin samples (42 dizygotic and 21 monozygotic) and three duplicate samples were removed from TwinsUK. The final sequence data set that was used for the association analyses comprises 3,621 samples samples (1,754 TwinsUK and 1,867 ALSPAC).

Data quality evaluation

Determination of sequencing accuracy against high read-depth exomes

We retrieved sequence data from a set of high read-depth exomes [30] recorded in 61 individuals having both whole-genome sequence and exome from the TwinsUK data set. Comparisons were carried out restricting the whole genome data to the bait regions that were used for the exome variant calling. The bait regions in these high read-depth sequence data samples covered 35,066,769 bp of sequence, and included a total of 82,998 sites called in the UK10K data. In total 74,621 exome sites (out of the 86,322 sites, or 86.4%) were shared with the UK10K low-genome samples.

Determination of site overlap with 1000 Genomes Project

To search for variant sites that are shared between UK10K Cohorts and the 1000GP only bi-allelic SNVs were taken into consideration. Variant overlap was assessed for allele frequencies (AF) bins calculated separately for the UK10K Cohorts and for the 1000GP set, in order to allow comparison between variant discovery in the two datasets. We anticipated at least 95% of the variants with AF > 1% would be found in the 1000GP data set [19].

Imputation from the combined UK10K + 1000 Genomes Panel

Genome-Wide SNP array data

For association tests, we also considered additional GWA data for each cohort. For ALSPAC, there were another 6,557 samples available, which were measured on Illumina HumanHap550 arrays[31]. For TwinsUK, there were another 2,575 samples that were unrelated to the sequence dataset (IBS>0.125) with

genotypes on Illumina HumanHap300 or Illumina Human610 arrays [32]. Both datasets passed QC criteria (gender check, heterozygosity, European ancestry, relatedness (ALSPAC) and zygosity (TwinsUK). Variants discovered through WGS of the TwinsUK and ALSPAC cohorts were imputed into the full GWAS genotyped cohorts increasing the sample size for single point association analysis up to 10,021 subjects.

The UK10K haplotype reference panel

The UK10K final release WGS data of 3,781 samples and 49,826,943 sites was used for creation of haplotype reference panel. For each chromosome, a summary file was first generated and merged with that of the 1000GP WGS data to identify multi-allelic sites, sites with inconsistent alleles with that of the 1000GP data, and singletons not existing in 1000GP. These sites were excluded to create a new set of VCF files, leaving 28,615,640 sites. The VCF-QUERY tool was used to convert the new VCF files into phased haplotypes and legend files for IMPUTE2. VCF files were converted to binary ped (bed) format and multi-allelic sites excluded, and files were then split into 3MB chunks with +/-250kb flanking regions. SHAPEIT v2 was used to rephrase the haplotypes. Phasing information from the SHAPEIT output was copied back to the original VCF files, with the phase removed for sites missing due to the MAF cut-off. The phased chunks were then recombined with vcf-phased-join from the vcftools package[5].

Prior to imputation, the two GWAS datasets (ALSPAC and TwinsUK) were pre-phased using SHAPEIT v2[27] to increase phasing accuracy. SHAPEIT v2 was also used for re-phasing the reference haplotypes provided by the UK10K project. Per the recommendation of the software, the mean size of the windows in which conditioning haplotypes are defined was set to 0.5MB, instead of 2MB used for pre-phasing GWAS. Due to the significantly higher number of variants in the WGS data, the re-phasing was conducted by 3MB chunk with 250kb buffering regions, rather than by whole chromosomes. Imputation was carried out on the same chunks with the same flanking regions as those of the reference panel using standard parameters with IMPUTE2.

SardiNIA WGS

The entire SardiNIA cohort was genotyped using four different Illumina chip arrays: the HumanOmniExpress, the Cardio-MetaboChip, the ImmunoChip and the HumanExome. These were resulted in quality-controlled 886,938 autosomal SNPs derived from all of the arrays. We also whole-genome sequenced 2,120 Sardinians at low pass (average 4-fold coverage), of which 1,122 were part of the SardiNIA project and 998 were individuals enrolled in case-control studies of Multiple Sclerosis and Type 1 Diabetes[35]

We then selected, from among sequenced samples, 1,488 unrelated individuals and created a reference panel to impute untyped markers in the whole cohort. Given the size of the reference panel, imputation was performed in haploid data using Minimac software, a modified version of the software MACH. Phased haplotypes were generated using MACH (--phase option) with 400 states and 30 rounds by subdividing the variants in 344 groups of 2,500 with an overlap of 500, and imputation was subsequently performed independently on each phased chunk. This procedure was used to perform imputation on all autosomal chromosomes using the Sardinian reference panel (SardSeq) as well as the 1000 Genomes Project reference panels (1000G), as suggested when planning to impute on very large imputation panels (for a description of the code, see http://genome.sph.umich.edu/wiki/Minimac:_1000_Genomes_Imputation_Cookbook

Supplementary References

1. Pruim, R.J. *et al.* LocusZoom: regional visualization of genome-wide association scan results. *Bioinformatics* **26**, 2336-7 (2010).
2. Porcu, E. *et al.* A meta-analysis of thyroid-related traits reveals novel loci and gender-specific differences in the regulation of thyroid function. *PLoS Genet* **9**, e1003266 (2013).
3. Carter, K.W., McCaskie, P.A. & Palmer, L.J. JLIN: a java based linkage disequilibrium plotter. *BMC Bioinformatics* **7**, 60 (2006).
4. Saviouk, V., Moreau, M.P., Tereshchenko, I.V. & Brzustowicz, L.M. Association of synapsin 2 with schizophrenia in families of Northern European ancestry. *Schizophr Res* **96**, 100-11 (2007).
5. Arnaud-Lopez, L. *et al.* Phosphodiesterase 8B gene variants are associated with serum TSH levels and thyroid function. *Am J Hum Genet* **82**, 1270-80 (2008).
6. Saraiva, M.J. Transthyretin mutations in hyperthyroxinemia and amyloid diseases. *Hum Mutat* **17**, 493-503 (2001).
7. Alves, I.L. *et al.* Thyroxine binding in a TTR Met 119 kindred. *J Clin Endocrinol Metab* **77**, 484-8 (1993).
8. Refetoff, S. *et al.* A new family with hyperthyroxinemia caused by transthyretin Val109 misdiagnosed as thyrotoxicosis and resistance to thyroid hormone--a clinical research center study. *J Clin Endocrinol Metab* **81**, 3335-40 (1996).
9. Fan, Y. *et al.* Molecular cloning, genomic organization, and mapping of beta 4GalT-VIb, a brain abundant member of beta 4-galactosyltransferase gene family, to human chromosome 18q12.1. *DNA Seq* **13**, 1-8 (2002).
10. Spector, T.D. & Williams, F.M. The UK Adult Twin Registry (TwinsUK). *Twin Res Hum Genet* **9**, 899-906 (2006).
11. Boyd, A. *et al.* Cohort Profile: the 'children of the 90s'--the index offspring of the Avon Longitudinal Study of Parents and Children. *Int J Epidemiol* **42**, 111-27.
12. Taylor, P.N., Razvi, S., Pearce, S.H. & Dayan, C.M. A review of the clinical consequences of variation in thyroid function within the reference range. *J Clin Endocrinol Metab* **98**, 3562-71 (2013).
13. Hadlow, N.C. *et al.* The relationship between TSH and free T4 in a large population is complex and nonlinear and differs by age and sex. *J Clin Endocrinol Metab* **98**, 2936-43 (2013).
14. Pilia, G. *et al.* Heritability of cardiovascular and personality traits in 6,148 Sardinians. *PLoS Genet* **2**, e132 (2006).
15. Naitza, S. *et al.* A genome-wide association scan on the levels of markers of inflammation in Sardinians reveals associations that underpin its complex regulation. *PLoS Genet* **8**, e1002480 (2012).
16. Traglia, M. *et al.* Heritability and demographic analyses in the large isolated population of Val Borbera suggest advantages in mapping complex traits genes. *PLoS One* **4**, e7554 (2009).
17. Walsh, J.P. *et al.* Thyrotropin and thyroid antibodies as predictors of hypothyroidism: a 13-year, longitudinal study of a community-based cohort using current immunoassay techniques. *J Clin Endocrinol Metab* **95**, 1095-104.
18. Li, H. *et al.* The Sequence Alignment/Map format and SAMtools. *Bioinformatics* **25**, 2078-9 (2009).
19. 1000 Genomes Project Consortium *et al.* A map of human genome variation from population-scale sequencing. *Nature* **467**, 1061-73 (2010).
20. Li, H. & Durbin, R. Fast and accurate short read alignment with Burrows-Wheeler transform. *Bioinformatics* **25**, 1754-60 (2009).
21. Li, H. A statistical framework for SNP calling, mutation discovery, association mapping and population genetical parameter estimation from sequencing data. *Bioinformatics* **27**, 2987-93 (2011).
22. Danecek, P. *et al.* The variant call format and VCFtools. *Bioinformatics* **27**, 2156-8 (2011).
23. Depristo, M.A. *et al.* A framework for variation discovery and genotyping using next-generation DNA sequencing data. *Nature Genetics* **43**, 491-498 (2011).
24. Mills, R.E. *et al.* Natural genetic variation caused by small insertions and deletions in the human genome. *Genome Res* **21**, 830-9 (2011).

25. Shin, S.Y. *et al.* An atlas of genetic influences on human blood metabolites. *Nat Genet* (2014).
26. Browning, S.R. & Browning, B.L. Rapid and accurate haplotype phasing and missing-data inference for whole-genome association studies by use of localized haplotype clustering. *American journal of human genetics* **81**, 1084-1097 (2007).
27. Delaneau, O., Marchini, J. & Zagury, J.F. A linear complexity phasing method for thousands of genomes. *Nat Methods* **9**, 179-81 (2012).
28. International HapMap 3 Consortium *et al.* Integrating common and rare genetic variation in diverse human populations. *Nature* **467**, 52-58 (2010).
29. Price, A.L. *et al.* Principal components analysis corrects for stratification in genome-wide association studies. *Nature Genetics* **38**, 904-909 (2006).
30. Williams, F.M. *et al.* Genes contributing to pain sensitivity in the normal population: an exome sequencing study. *PLoS Genet* **8**, e1003095 (2012).
31. Bonnelykke, K. *et al.* Meta-analysis of genome-wide association studies identifies ten loci influencing allergic sensitization. *Nat Genet* **45**, 902-6 (2013).
32. Soranzo, N. *et al.* Meta-analysis of genome-wide scans for human adult stature identifies novel Loci and associations with measures of skeletal frame size. *PLoS Genetics* **5**, e1000445 (2009).
33. Paternoster, L. *et al.* Genetic determinants of trabecular and cortical volumetric bone mineral densities and bone microstructure. *PLoS Genet* **9**, e1003247 (2013).
34. Suhre, K. *et al.* Human metabolic individuality in biomedical and pharmaceutical research. *Nature* **477**, 54-60 (2011).
35. Sanna, S. *et al.* Variants within the immunoregulatory CBLB gene are associated with multiple sclerosis. *Nat Genet* **42**, 495-7 (2010).
